# Interactome Analysis Identifies the Role of BZW2 in Promoting Endoplasmic Reticulum-Mitochondria Contact and Mitochondrial Metabolism

**DOI:** 10.1016/j.mcpro.2023.100709

**Published:** 2023-12-26

**Authors:** George Maio, Mike Smith, Ruchika Bhawal, Sheng Zhang, Jeremy M. Baskin, Jenny Li, Hening Lin

**Affiliations:** 1 Department of Chemistry and Chemical Biology, Cornell University, Ithaca, New York, USA; 2 Proteomics and Metabolomics Facility, Cornell University, Ithaca, New York, USA; 3 Weill Institute for Cell and Molecular Biology, Cornell University, Ithaca, New York, USA; 4 Howard Hughes Medical Institute, Cornell University, Ithaca, New York, USA; 5 Department of Molecular Biology and Genetics, Cornell University, Ithaca, New York, USA

**Keywords:** BZW2, MERCS, interactome, SILAC, mitochondria, endoplasmic reticulum, metabolism, AMPK, VDAC2, BCAP31, ATP, lipidomics, lipid droplet, calcium flux

## Abstract

Understanding the molecular functions of less-studied proteins is an important task of life science research. Despite reports of basic leucine zipper and W2 domain-containing protein 2 (BZW2) promoting cancer progression first emerging in 2017, little is known about its molecular function. Using a quantitative proteomic approach to identify its interacting proteins, we found that BZW2 interacts with both endoplasmic reticulum (ER) and mitochondrial proteins. We thus hypothesized that BZW2 localizes to and promotes the formation of ER-mitochondria contact sites and that such localization would promote calcium transport from ER to the mitochondria and promote ATP production. Indeed, we found that BZW2 localized to ER-mitochondria contact sites and that BZW2 knockdown decreased ER-mitochondria contact, mitochondrial calcium levels, and ATP production. These findings provide key insights into molecular functions of BZW2, the potential role of BZW2 in cancer progression, and highlight the utility of interactome data in understanding the function of less-studied proteins.

## Highlights

• BZW2 interactome identifies both ER and mitochondrial proteins.
• BZW2 promotes mitochondria-ER contact, mitochondrial calcium, and ATP production.
• Lack of BZW2 leads to low ATP levels, AMPK activation, and increased FAO.
• Interactome analysis aids in the understanding of less-studied proteins.

## Graphical abstract

**Figure:**
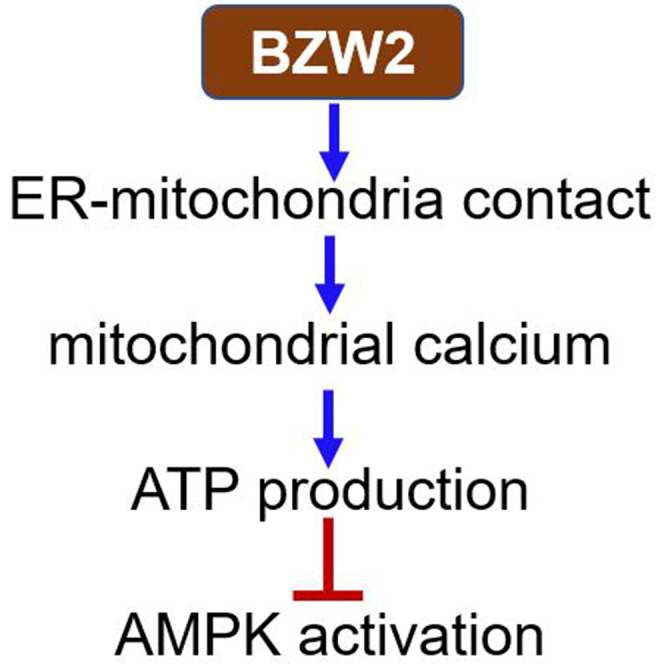

## In Brief

Maio *et al.* presents a comprehensive and quantitative interactome of BZW2, a lesser-studied protein associated with the progression of many human cancers. This study uncovers a novel molecular function of BZW2 including promoting mitochondria-ER contact, mitochondrial calcium, and ATP production. The finding provides molecular insights into the roles of BZW in normal and cancer cells and highlights the importance of interactome studies in understanding the function of less studied proteins.

Understanding the functions of individual proteins is central to understanding the molecular foundations of life and disease. However, a stark inequity exists with respect to our understanding of the proteome at the level of individual proteins. Whereas substantial research efforts are devoted to proteins with well-known functions, minimal research efforts focus on proteins with few or no known functions (1). This divergence is understandable due to the inherent difficulty of studying proteins with no known functions. However, many of these proteins may still play significant roles in biological processes and disease progression. Thus, there is an urgent need to reveal functional information for the substantial fraction of the proteome that is poorly studied.

Since its initial characterization in 2011, basic leucine zipper and W2 domain-containing protein 2 (BZW2, also known as 5MP1) has been shown to play a role in critical biological processes such as promoting cell cycle progression (2, 3, 4, 5, 6, 7, 8), inhibiting apoptosis (3, 5, 6, 8, 9, 10), and altering translation initiation start sites (2, 11, 12, 13, 14, 15). Its oncogenic role is beginning to emerge with reports of BZW2 promoting a variety of cancers including acute myeloid leukemia, multiple myeloma, throat, bone, bladder, colorectal, pancreatic, and liver cancer (2, 3, 4, 5, 6, 7, 8, 9, 10, 16, 17, 18, 19, 20). Furthermore, BZW2 promotes established oncogenic signaling pathways such as RAS/MAPK and PI3K/AKT (4, 8, 16) as well as increases expression levels of the oncoprotein c-Myc (2, 5, 21). However, little is known about the molecular mechanisms behind these observations. Despite BZW2’s clear involvement in disease progression, it currently remains an understudied protein. A PubMed search for BZW2 conducted in February 2023 returned 28 publications, a stark contrast to the well-known oncoprotein RAS which returned >94,000 publications. Consequently, understanding of BZW2 molecular functions is only in its infancy and critical information such as its cellular localization, interacting partners, or how its biomolecular interactions and behavior influence cellular functions remain largely unexplored.

To gain insights into the function of proteins with few known molecular functions, we have previously relied on identifying interacting partners through a quantitative interactome analysis. For instance, we have previously harnessed interactome data to identify substrate proteins for N-terminal glycine myristoyltransferase and discover the enzyme that reduces the iron-containing protein Dph3 (22, 23). Whereas some interacting proteins of BZW2, such as eukaryotic initiation factors 2 and 3 (eIF2 and eIF3), have been reported (11), a comprehensive and quantitative list of BZW2-binding partners has yet to be reported. Therefore, we used a quantitative proteomic approach to identify the BZW2 interactome. Analysis of these data led us to formulate and test a hypothesis about the molecular function of BZW2 as a new regulator of ER-mitochondria contact sites and mitochondrial metabolism. Our study not only provides key insights into the molecular function of BZW2 but also suggests that high-quality interactome data could play an important role in elucidating the functions of understudied proteins.

## Experimental procedures

### Antibodies and Common Reagents

**Table:** 

Key resources table				
Reagent type (species) or resource	Designation	Source or reference	Identifiers	Additional information
Cell line (human)	HEK-293T	ATCC	CRL-3216	
Cell line (human)	HEK-293	ATCC	CRL-1573	
Cell line (human)	A549	ATCC	CCL-185	
Cell line (human)	AsPC-1	ATCC	CRL-1682	
Cell line (human)	HCT116	ATCC	CCL-247	
Cell line (human)	HT-29	ATCC	HTB-38	
Cell line (human)	SW620	ATCC	CCL-227	
Cell line (human)	MDA-MB-231	ATCC	CRM-HTB-26	
Cell line (human)	Flag-BZW2 HEK-293	This paper	N/A	Generation described in cloning, transfection, and transduction
Cell line (human)	BZW2 HEK-293	This paper	N/A	Generation described in cloning, transfection, and transduction
Cell line (human)	shLuciferase HEK-293	This paper	N/A	Generation described in cloning, transfection, and transduction
Cell line (human)	shBZW2 HEK-293	This paper	N/A	Generation described in cloning, transfection, and transduction
Transfected construct	pLKO.1_shLuciferase	Sigma-Aldrich	SHC007	
Transfected construct (human)	pLKO.1_BZW2 sh1	Sigma-Aldrich	TRCN0000144193	
Transfected construct (human)	pLKO.1_BZW2 sh2	Sigma-Aldrich	TRCN0000140132	
Transfected construct (human)	pLKO.1_BZW2 sh3	Sigma-Aldrich	TRCN0000140053	
Transfected construct (human)	pLKO.1_BZW2 sh4	Sigma-Aldrich	TRCN0000430209	
Transfected construct (human)	pLKO.1_BZW2 sh5	Sigma-Aldrich	TRCN0000145083	
Transfected construct (human)	pCDH_Flag-BZW2	this paper	N/A	Cloning described in cloning, transfection, and transduction
Transfected construct (human)	pCDH_BZW2	this paper	N/A	Cloning described in cloning, transfection, and transduction
Transfected construct (human)	pCDH_Flag-BZW2ΔW2	This paper	N/A	Cloning described in cloning, transfection, and transduction
Transfected construct (human)	pCDH_Flag-W2(BZW2)	This paper	N/A	Cloning described in cloning, transfection, and transduction
Transfected construct (human)	CMV5_Flag-BZW2	This paper	N/A	Cloning described in cloning, transfection, and transduction
Transfected construct (human)	CMV5_Flag-RalA Q72L	This paper	N/A	Cloning described in cloning, transfection, and transduction
Transfected construct (human)	CMV5_Flag-RalA S28N	This paper	N/A	Cloning described in cloning, transfection, and transduction
Transfected construct (human)	CMV5_Flag-RalB Q72L	This paper	N/A	Cloning described in cloning, transfection, and transduction
Transfected construct (human)	CMV-Mito4x-GCaMP6f	Addgene	#127870	
Antibody	Anti-BCAP31 (rabbit polyclonal)	Proteintech	11200-1-AP	WB (1:1000)
Antibody	Anti-VDAC2 (rabbit polyclonal)	Proteintech	11663-1-AP	WB (1:1000)
Antibody	Anti-VDAC1/2 (rabbit polyclonal)	Proteintech	10866-1-AP	IF (1:200)
Antibody	Anti-MIC60/Mitofilin (rabbit polyclonal)	Proteintech	10179-1-AP	WB (1:1000)
Antibody	Anti-Hexokinase II (rabbit polyclonal)	Proteintech	22029-1-AP	WB (1:1000) IF(1:100)
Antibody	Anti-FACL4 (rabbit polyclonal)	Proteintech	22401-1-AP	WB (1:1000)
Antibody	Anti-BZW2 (rabbit polyclonal)	Abcam	ab96682	WB (1:1000) IF(1:100)
Antibody	Anti-AGK (rabbit polyclonal)	Abcam	ab137616	WB (1:1000)
Antibody	Anti-GRP75 (rabbit polyclonal)	Abclonal	A0558	WB (1:1000)
Antibody	Anti-GAPDH (mouse monoclonal)	Santa Cruz Biotechnology	SC-47724 HRP	WB (1:2000)
Antibody	Anti-Actin (mouse monoclonal)	Santa Cruz Biotechnology	SC-47778 HRP	WB (1:2000)
Antibody	Anti-AMPKα (rabbit polyclonal)	Cell Signaling Technology	5831	WB (1:1000)
Antibody	Anti-p-AMPKα (Thr172) (rabbit polyclonal)	Cell Signaling Technology	2535	WB (1:1000)
Antibody	Anti-Pan AKT (rabbit polyclonal)	Cell Signaling Technology	4691	WB (1:1000)
Antibody	Anti-p-AKT (T308) (rabbit polyclonal)	Cell Signaling Technology	2965	WB (1:1000)
Antibody	Anti-p-AKT (S473) (rabbit polyclonal)	Cell Signaling Technology	9271	WB (1:1000)
Antibody	Anti-c-MYC (rabbit polyclonal)	Cell Signaling Technology	5605	WB (1:1000)
Antibody	Anti-Calnexin (rabbit polyclonal)	Cell Signaling Technology	2679	WB (1:1000) IF(1:100)
Antibody	Anti-αTubulin (mouse polyclonal)	Cell Signaling Technology	3873	WB (1:1000)
Antibody	Anti-FLAG M2 conjugated with HRP	Sigma-Aldrich	A8592	WB (1:5000)
Antibody	Anti-FLAG M2 affinity gel	Sigma-Aldrich	A2220	IP (10 μl slurry/1 mg total protein)
Chemical compound, drug	L-lysine	Sigma-Aldrich	L9037	
Chemical compound, drug	L-arginine	Sigma-Aldrich	A8094	
Chemical compound, drug	[13C6, 15N2]-L-lysine	Sigma-Aldrich	608,041	
Chemical compound, drug	[13C6, 15N4]-L-arginine	Sigma-Aldrich	608,033	
Chemical compound, drug	Protease inhibitor cocktail	Sigma-Aldrich	P8340	
Chemical compound, drug	Halt phosphatase inhibitor cocktail	Sigma-Aldrich	78,428	
Software	Fiji	N/A	https://fiji.sc/	
Software	Seahorse Wave 2.6 Desktop	Agilent Technologies	https://www.agilent.com/en	
Software	GraphPad Prism	GraphPad Software	https://www.graphpad.com/	
Other	Ponceau S	Sigma-Aldrich	P3504	
Other	Rhod-2 AM	Cayman Chemical	CAT#: 19355 CAS: 45037-81-6	
Other	Seahorse XF Real-Time ATP Rate Assay Kit	Agilent Technologies, Inc	103592-100	
Other	Seahorse Glycolytic Rate Assay Kit	Agilent Technologies, Inc	103344-100	
Other	QuantiChrom Calcium Assay Kit	BioAssay Systems	DICA-500	
Other	Saponin	TCI America	S0019	
Other	Qproteome Mitochondria Isolation Kit	Qiagen	37612	
Other	CellTiter-Glo 2.0 Cell Viability Assay	Promega	G9241	
Other	Sequencing grade modified trypsin	Promega	V5111	
Other	Polyethylenimine hydrochloride	PolySciences, Inc	24765-1	
Other	Clarity Max Western ECL Substrate	Bio-Rad Laboratories	1705062	
Other	Pierce ECL Western Blotting Substrate	Thermo Fisher Scientific	32106	
Other	Pierce BCA Protein Assay Kit	Thermo Fisher Scientific	23225	
Other	Paraformaldehyde solution, 4% in PBS	Thermo Fisher Scientific	AAJ19943K2	
Other	MitoTracker Deep Red FM	Thermo Fisher Scientific	M22426	
Other	Fluoromount-G	Thermo Fisher Scientific	00-4958-02	
Other	FlexAble CoraLite Plus 405	Proteintech	KFA006	
Other	FlexAble CoraLite Plus 488	Proteintech	KFA001	
Other	FlexAble CoraLite Plus 647	Proteintech	KFA003	

### Cell Culture

Human Embryonic Kidney (HEK) 293 and 293T cells were cultured in Dulbecco's Modified Eagle Medium (Thermo Fisher Scientific) with 10% heat-inactivated fetal bovine serum (Thermo Fisher Scientific). A549 and AsPC-1 cells were cultured in RPMI 1640 Medium (Thermo Fisher Scientific) supplemented with 10% heat inactivated fetal bovine serum. All cell lines obtained from ATCC were not further authenticated after purchase from ATCC. All the cell lines have been tested for *mycoplasma* contamination and showed no *mycoplasma* contamination.

### Cloning, Transfection, and Transduction

Human BZW2 (amplified using cDNA from Transomic) was inserted into pCMV5 and pCDH vector with an N-terminal Flag tag. Tag-free human BZW2 was inserted into pCDH vector. Human RalA and RalB containing single point mutations Q72L and S28N with an N-terminal Flag tag were inserted into pCMV5 vector. Transient transfections were performed using PEI hydrochloride according to manufacturer’s protocol. Lentiviruses were generated by cotransfection of luciferase or BZW2 shRNA in pLKO.1 vector or Flag-tagged BZW2/tag-free BZW2 in pCDH with pCMV-ΔR8.2 and pMD2.G into HEK293T cells. Media containing viral particles were filtered (0.42 μm) and stored in −80 °C. Viral transductions were performed by adding lentivirus/fresh media (3:1, v:v) containing 6 μg/μl polybrene to cells and incubating for 6 h to overnight at 37 °C in 5% CO_2_. Media was then replaced with fresh culture media and grown for 48 to 72 h before collecting or being selected for stable cell line generation using 6 μg/μl puromycin.

### Immunoprecipitation

Cells were collected and lysed in 1% NP40 lysis buffer (1% NP40, 25 mM Tris–HCl pH 7.4, 150 mM NaCl and 10% glycerol) with protease inhibitor cocktail (1:100 dilution) and phosphatase inhibitor cocktail (1:100 dilution) on ice for 30 min. After centrifuging at 17,000*g* for 15 min, supernatant (total lysates) was collected for FLAG immunoprecipitation using anti-FLAG M2 affinity gel following manufacturer’s protocol. The affinity gel was washed three times with NP40 washing buffer (0.2% NP40, 25 mM Tris–HCl pH 7.4, and 150 mM NaCl). To detect the interacting proteins, the affinity gel was heated at 95 °C for 10 min in 2x protein loading buffer, followed by Western blot analysis.

### Western Blot

Proteins were resolved by 12% SDS-PAGE and transferred to a polyvinylidene difluoride or nitrocellulose membrane. The membrane was blocked using 5% bovine serum albumin (BSA) or nonfat milk in TBST (0.1% Tween-20 in TBS solution) at room temperature for 60 min. Antibodies were diluted in fresh 5% BSA or nonfat milk in TBST and then incubated according to the manufacturer’s protocol, either 1 h at room temperature or overnight at 4 °C. After washing the membrane three times with TBST, the secondary antibody (1:5000 dilution in 5% BSA or nonfat milk in TBST) was added and then incubated at room temperature for 1 h. Following three more washes with TBST, the chemiluminescence signal was recorded after developing in ECL Plus Western Blotting Substrate (Thermo Fisher Scientific) using ChemiDoc MP Imaging System (Bio-Rad).

### SILAC Experimental Design

SILAC sample preparation and LC-MS/MS analysis were performed following previously published methods (24, 25). Briefly, four total SILAC samples were prepared and analyzed by LC-MS/MS consisting of four BZW2 biological replicates. The biological replicates were done such that one was a ‘forward’ experiment and the second was a ‘reverse’ experiment, each performed in duplicate (two ‘forward’ and two ‘reverse’ experiments). In the forward experiment, HEK293 cells stably expressing tag-free BZW2 were cultured in light media (containing light isotope–labeled Lys or Arg) and HEK293 stably expressing Flag-tagged BZW2 were cultured in heavy media (containing heavy isotope–labeled Lys and Arg). Enrichment of both the Flag-tagged BZW2 (heavy) and tag-free BZW2 (light) was performed using anti-FLAG M2 affinity gel (Flag beads) following the manufacturer’s protocol. Before carrying out the enrichment step, we ensured that equal amount of protein input was used for each sample (supplemental Fig. S1*A*). After enrichment, the Flag beads were washed 3x using NP40 wash buffer. Both the heavy and light flag beads were then reconstituted in 500 μl of NP40 washing buffer and combined before protein elution. In the reverse experiment, HEK293 cells stably expressing Flag-tagged BZW2 were cultured in light media and HEK293 stably expressing tag-free BZW2 were cultured in heavy media. The procedure for enrichment, washing, mixing, and elution were identical to the forward experiment. Only proteins identified in both forward and reverse SILAC experiments were kept for further validation.

### Protein Digestion and NanoLC-MS/MS Analysis

To elute FLAG-tagged protein with its interacting proteins, the affinity gel was heated at 95 °C for 10 min in 1% SDS elution buffer (1% SDS, 25 mM Tris–HCl pH 7.4, and 150 mM NaCl), followed by methanol/chloroform protein precipitation. The protein pellets were denatured in 6 M urea, 10 mM DTT, and 50 mM Tris–HCl pH 8.0 at room temperature for 1 h. The proteins were alkylated by incubating with 40 mM iodoacetamide at room temperature for 1 h. DTT was then added to stop alkylation at room temperature for 1 h. After diluting the protein sample seven times with 50 mM Tris–HCl pH 8.0 and 1 mM CaCl2, 1 μg of trypsin was added and incubated with the protein at 37 °C for 18 h. TFA (0.1% in water) was added to quench the trypsin digestion, followed by desalting using a Sep-Pak C18 cartridge. Lyophilized peptides were dissolved in 2% acetonitrile (ACN) with 0.5% formic acid (FA) estimated at 0.1 μg/μl for nanoLC-ESI-MS/MS analysis on UltiMate3000RSLCnano-Orbitrap Fusion system (Thermo Fisher Scientific). The reconstituted peptides were injected into an Acclaim PepMap nano Viper C18 trap column (5 μm, 100 μm i.d. × 2 cm, Thermo Fisher Scientific) at 20 μl/min flow rate for rapid loading and separated on a PepMap C18 RP nano column (5 μm, 75 μm i.d. × 25 cm, Thermo Fisher Scientific) at 35 °C. The flow rate was set as 0.3 μl/min. The gradient was set as follows: 4 to 5% ACN with 0.1% FA (0–3 min), 5 to 35% ACN with 0.1% FA (3–123 min), 35 to 90% ACN with 0.1% FA (123–131 min), 90% ACN with 0.1% FA (131–140 min), 90 to 4% ACN with 0.1% FA (140–141 min), and 4% ACN (141–165 min). The Orbitrap Fusion was operated in positive ion mode with spray voltage 1.7 kV and source temperature 275 °C under 3 s “Top Speed” data-dependent acquisition analysis. In data-dependent acquisition analysis, a survey MS scan was acquired from m/z 375 to 1575 at a resolving power of 120,000 using an FT mass analyzer followed by data-dependent CID ion trap MS/MS scans for precursor peptides with multiple charged ions above a threshold ion count of 20,000 and normalized collision energy of 30% along with an automated gain control target of 10,000.

### Mass Spectrometry Data Analysis and Statistical Rationale

Raw files generated were analyzed with Proteome Discoverer 2.4 (PD 2.4 Thermo Fisher Scientific) using Sequest HT against the NCBI human database (April 2019, 81,268 entries). The database search was performed under a search workflow with the “Precursor Ions Quantifier” node for SILAC 2plex (Arg10, Lys8) quantitation. Trypsin (Full) was set as the enzyme, allowing for two maximum missed cleavages. All searches were performed with carbamidomethylation (57.021 Da) of cysteines as a static modification whereas methionine oxidation (15.995 Da), protein N-terminal acetylation (42.011 Da), heavy labeled lysine (8.014 Da), and heavy labeled arginine (10.008 Da) were set as dynamic modifications. The mass tolerance for precursor ions was set at 10 ppm and the mass tolerance for fragment ions was set at 0.6 Da. Minimum peptide length of six amino acids was required for all identifications. Percolator was used as the false discovery rate calculator and all peptides were filtered at the strict target false discovery rate of 0.01 from a reversed sequence database. Proteins had to be identified by a minimum of two peptides to be counted. The SILAC 2-plex quantification method within PD 2.4 was used with unique + razor peptides only to calculate the heavy/light ratios using pairwise ratio based for all identified proteins without normalization. Each peptide H/L ratio was determined by a regression model fitted to all isotopic peaks within all scans during which the peptide eluted in. Each protein H/L ratio was determined as the median of all peptides assigned to that protein. Proteins with a H/L ratio ≥4.0 in the forward sample and a H/L ratio ≤0.4 in the reverse sample for at least three of the four biological replicates were identified as BZW2 interactors (*e.g.*, forward H/L ≥ 4.0 in two replicates and reverse H/L ≤ 0.4 in one replicate or forward H/L ≥ 4.0 in one sample and reverse H/L ≤ 0.4 in two replicates).

### Lipidomics Sample Preparation

Stable shLuciferase or shBZW2 HEK293 cells were equally seeded in five separate 100 mm cell culture dishes and grown to ∼80% confluency (∼2 × 10^6^ cells). Cells were collected in separate 1.5 ml Eppendorf tubes, washed three times using 1x PBS on ice, then snap-frozen using liquid nitrogen, and stored in −80 °C for future processing.

To lyse the cells, 100 μl Optima H_2_O was added and vortexed to resuspend pellet, then transferred to High GForce 1.5 ml Eppendorf (VWR #20170-038). Original tube was rinsed with an additional 25 μl optima H_2_O and transferred to High G-force tube. Total vol ∼130 μl Optima water/cell pellet was sonicated on ice for 10 min.

To the ∼130 μl lysed cells, 30 μl of internal standard plus 345 μl cold chloroform:MeOH (1:2 v/v) was added and vortexed for 10 s and then sonicated on ice for 10 min. An additional 125 μl of 100% chloroform was added and vortexed for 10 s. After sitting for 1 min, an additional 125 μl Optima H_2_O was added and vortexed for 10 s and then left to equilibrate for 10 min at room temperature. Samples were then centrifuged at 18,000*g* for 10 min at 4 °C. Using a long gel loading tip, 220 μl of lower phase was transferred into a clean glass culture tube and then evaporated to dryness in a speed vac. Capped glass tube was then stored at −20 °C until ready to reconstitute for LC-MS/MS analysis.

The internal standard mixture spiked into each sample before extraction consisted of 25 μg/ml of TG (15:0)_3_, PG (14:0)_2_, PS (16:0)_2_, Cer(d18:1_12:0), ChE(17:0), and 5 μg/ml LPC(18:1D7), PC(18:1D7_15:0) in DCM/MeOH (2:1).

### Lipidomics LC-MS/MS Analysis

Immediately prior to analysis, the lipid extract was diluted with isopropanol/acetonitrile/water (2:1:1, 100 μl). The lipid samples were analyzed by Vanquish UHPLC coupled with Q-Exactive HF system (QE-HF, Thermo Fisher Scientific) as described previously (26). The Vanquish UHPLC conditions were as follows: column: Accucore C30, 2.6 μm column (2.1 mm id × 150 mm). Flow rate: 260 μl/min. Column temperature: 55 °C. Autosampler tray temperature: 4 °C. Injection volume: 2 μl. Solvents: A) 60% ACN, 40% H_2_O, 10 mM ammonium formate with 0.1% formic acid; B) 90% isopropyl alcohol, 10% ACN, 10 mM ammonium formate with 0.1% formic acid. The QE-HF was operated as follows: ESI voltage: 4 kV. Sheath gas flow rate: 50 (arbitrary unit). Aux gas flow rate: 5. Sweep gas flow rate: 1. Capillary temperature: 320 °C. S-Lens RF level: 50. Aux gas heater temperature: 350 °C. Both positive and negative modes were used for acquisition of lipidomics datasets.

### Lipidomics Data Analysis

Nontargeted lipidomics were done comparing two different groups (shLuciferase and shBZW2 HEK293 cells). Data Processing Thermo Scientific LipidSearch software version 4.2 was used for lipid identification, with the following workflow as described previously (26).

First, the individual data files were searched for product ion MS/MS spectra of lipid precursor ions. MS/MS fragment ions were predicted for all precursor adduct ions measured within ±5 ppm. The product ions that matched the predicted fragment ions within a ±5 ppm mass tolerance were used to calculate a match-score, and those candidates providing the highest quality match were determined.

Next, the search results from the individual positive or negative ion files from each sample group were aligned within a retention time window (±0.1 min) and the data were merged for each annotated lipid.

The annotated lipids were then filtered to reduce false positives. Data were normalized using the spiked internal standards for the different classes and annotated lipids were exported to an Excel spreadsheet.

Four hundred fifty two lipid species were identified in positive ion mode (excluding isomers after filtering the data) on the Lipidmaps, and three hundred eighteen lipid species were identified in negative ion mode (after filtering the data) on the Lipidmaps The largest number of lipid category in positive and negative ion modes corresponded to TG and PE, respectively. All lipid species were then grouped into their respective lipid class (*e.g.*, PC, PG, TG). The total abundance of each lipid class was calculated as the sum of the lipid species abundance within that respective class. A Student *t* test was performed on the total abundance of each lipid class for all five shLuciferase and shBZW2 samples. Fold change of shBZW2/shLuciferase on total abundance for each lipid class with a *p*-value ≤0.05 was calculated and the log2(FC) was determined.

### Sample Preparation for Transient BZW2 KD and Overexpression

HEK293, AsPC-1, and A549 were seeded into 10-cm cell culture dishes to ∼70% confluency. Cells were then transduced with either shLuciferase or shBZW2 lentivirus overnight. Cells were then split equally into two fresh 10-cm cell culture dishes and grown for 24 h. Cells were then transfected with either empty pCDH vector or pCDH vector containing tag-free BZW2 and allowed to grow for 24 h. Cells were then seeded into experimental samples with the remaining cells collected and stored in −80 °C for future WB analysis.

### Statistical Analysis for Transient BZW2 KD and Re-expression

Differences between control knockdown (KD), shBZW2, and shBZW2+BZW2 in all transient KD and overexpression experiments were examined using an ordinary one-way ANOVA with no matching or pairing, comparing the mean of each column with the mean of every other one (Brown-Forsythe and Welch’s ANOVA test). The *p*-values were indicated (∗*p* < 0.05, ∗∗*p* < 0.01, ∗∗∗*p* < 0.001, and ∗∗∗∗*p* < 0.0001).

### BZW2-ER-Mitochondria Immunofluorescence

AsPC-1 cells were seeded (∼2.5 × 10^5^ cells) into 35 mm glass bottom dishes (MatTek) and allowed to adhere overnight. Media was removed and cells were fixed using 4% paraformaldehyde (Sigma). Cells were then washed twice with 1x PBS, permeabilized using 0.2% Triton-X in PBS for 10 min, and washed three times with 1x PBS. Cells were then blocked using imaging buffer (5% BSA, 0.1% Saponin in PBS) for 1 h at RT protected from light. Samples were then incubated with imaging buffer containing primary Calnexin (CST, Rabbit) antibody (1:100) conjugated with FlexAble CoraLite Plus 405 (Proteintech), primary BZW2 (Abcam, Rabbit) antibody (1:100) conjugated with FlexAble CoraLite Plus 488, and primary VDAC (CST, rabbit) antibody (1:200) conjugated with FlexAble CoraLite Plus 647 (Proteintech) and then left to incubate for 2 h at room temperature. All FlexAble CoraLite kits were used according to manufacturer’s protocol. Cells were then washed three times using 1× PBS and two drops of Fluoromount-G were added. Samples were stored at 4 °C protected from light until imaged under 63× magnification using a Zeiss LSM710 inverted confocal microscope.

### ER-Mitochondria Immunofluorescence

shLuciferase/shBZW2 lentivirus transduced and pCDH/tag-free BZW2 transfected HEK293, AsPC-1, and A549 cells were seeded (∼2.5 × 10^5^ cells) into 35 mm glass bottom dishes (MatTek) and allowed to adhere overnight. Media was removed and cells were fixed using 4% paraformaldehyde (Sigma). Cells were then washed twice with 1x PBS, permeabilized using 0.2% Triton-X in PBS for 10 min, and washed three times with 1x PBS. Cells were then blocked using imaging buffer (5% BSA, 0.1% Saponin in PBS) for 1 h at RT. Samples were then incubated with imaging buffer containing primary VDAC (CST, rabbit) antibody (1:200) conjugated with FlexAble CoraLite Plus 647 (Proteintech) and primary Calnexin (CST, Rabbit) antibody (1:100) conjugated with FlexAble CoraLite Plus 405 (Proteintech) for 2 h at room temperature. All FlexAble CoraLite kits were used according to manufacturer’s protocol. Cells were then washed three times using 1x PBS and two drops of Fluoromount-G were added. Samples were stored at 4 °C protected from light until imaged under 63× magnification using a Zeiss LSM710 inverted confocal microscope.

### Immunofluorescence Colocalization Data Analysis

Images were analyzed using Fiji ImageJ software where background was removed using rolling ball radius of 25 pixels. Colocalization was analyzed using the ‘EZColocalization’ (27) plugin with default thresholds for all channels. Regions of interest (ROI’s) were drawn around individual cells using the freehand tool and Pearson correlation coefficient (PCC), Spearman’s rank correlation coefficient (SRCC), and Manders' correlation coefficient (M1, M2, or M3) were determined for each ROI (A549: CtrlKD + vector n = 109, shBZW2 + vector n = 104, shBZW2 + BZW2 n = 103; AsPC-1: CtrlKD + vector n = 108, shBZW2 + vector n = 102, shBZW2 + BZW2 n = 101, no treatment for M3 analysis n = 121; HEK293: CtrlKD + vector n = 106, shBZW2 + vector n = 116, shBZW2 + BZW2 n = 105).

### Mitochondrial Calcium Flux Using Rhod-2AM and Mito4x-GCaMP6f

shLuciferase/shBZW2 lentivirus transduced and pCDH/tag-freeBZW2 transfected HEK293 cells were seeded (∼2.5 × 10^5^ cells for Rhod-2AM, ∼1.0 × 10^5^ cells for Mito4x-GCaMP6f) into 35 mm glass bottom dishes (MatTek) and allowed to adhere overnight. For Mito4x-GCaMP6f samples, cells were transfected with 1.5 μg Mito4x-GCaMP6f and allowed to incubate overnight. For Rhod-2AM samples, cells were washed once with 1x PBS and treated with Hanks Balanced Salt Solution containing 1 μM Rhod-2AM (Invitrogen) or 1 μM Rhod-2AM reduced with NaBH_4_ (Rhod-2AM_r_) and incubated in 37 °C for 30 min. The solution was then replaced with live cell imaging media (Invitrogen) and incubated for 15 min in 37 °C until imaged under 40x magnification using a Zeiss LSM710 inverted confocal microscope. After initial fluorescence intensities were captured for a subset of cells, 5 μM of ionomycin was added to fully saturate fluorescence signal. Maximum fluorescence intensities were then captured for the same cells.

### Mitochondrial Calcium Flux Data Analysis

Images were analyzed using Fiji ImageJ software. ROI’s were drawn around individual cells using the freehand tool. Initial fluorescence intensities were labeled as F_0_, while fully saturated fluorescence intensities were labeled as F_MAX_. Calcium flux was then calculated using F_MAX_/F_0_ (Mito4x-GCaMP6f: CtrlKD + vector n = 31; shBZW2 + vector n = 25; shBZW2 + BZW2 n = 22. Rhod-2AM_r_: CtrlKD + vector n = 61; shBZW2 + vector n = 79; shBZW2 + BZW2 n = 83. Rhod-2AM: CtrlKD + vector n = 77; shBZW2 + vector n = 69; shBZW2 + BZW2 n = 84).

### Basal Mitochondrial Calcium Imaging with Rhod-2 AM

shLuciferase/shBZW2 lentivirus transduced and pCDH/tag-free BZW2 transfected HEK293, AsPC-1, and A549 cells were seeded (∼2.5 × 10^5^ cells) into 35 mm glass bottom dishes (MatTek) and allowed to adhere overnight. Cells were then washed once with 1x PBS and treated with Hanks Balanced Salt Solution containing 1 μM Rhod-2 AM (Invitrogen) and incubated in 37 °C for 30 min. The solution was then replaced with live cell imaging media (Invitrogen) and incubated for 15 min in 37 °C until imaged under 40× magnification using a Zeiss LSM710 inverted confocal microscope.

### Basal Mitochondrial Calcium Rhod-2 AM Data Analysis

Images were analyzed using Fiji ImageJ software. ROI’s were drawn around individual cells using the freehand tool. Area and mean gray value were measured for each ROI. Calcium levels were determined using (mean/area) as a percentage.

### Oil Red O Staining

Media of the shLuciferase/shBZW2 lentivirus transduced and pCDH/tag-freeBZW2 transfected HEK293, AsPC-1, and A549 cells were seeded (∼2.5 × 10^5^ cells) into 35 mm glass bottom dishes (MatTek) and allowed to adhere overnight. Media was then removed and replaced with 10% formalin in PBS for 10 min at RT. Solution was replaced with fresh 10% formalin in PBS and incubated for 1 to 2 h at RT. Cells were then washed using 60% isopropanol and allowed to dry completely. Oil Red O solution (OilRedO:dH_2_O | 6:4) (filtered 0.2 μm) was added to cells and incubated for 10 min at RT. Solution was removed and quickly washed four times with excess dH_2_O. Water was removed and allowed to dry before imaging under 20× magnification using color brightfield on a Cytation 5 (Biotek).

### Oil Red O Data Analysis

Images were converted to RGB using Fiji ImageJ software. Rei/entropy threshold was used with the red channel set to 255. Particles were analyzed for the entire image measuring the number of particles and total area. Cell number was then counted using the ‘Cell Counter’ plugin (HEK293: CtrlKD n = 163, shBZW2 n = 176, shBZW2+BZW2 n = 123; AsPC-1: CtrlKD n = 185, shBZW2 n = 144, shBZW2+BZW2 n = 233; A549: CtrlKD n = 220, shBZW2 n = 201, shBZW2+BZW2 n = 222). Lipid droplet (LD) size was obtained by total area/number of particles. LD level was determined using total area/cell count.

### ATP Production

shLuciferase/shBZW2 lentivirus transduced and pCDH/tag-free BZW2 transfected HEK293 cells were seeded (∼3.0 × 10^4^ cells) in five separate wells of a 96-well cell culture plate and allowed to adhere overnight. ATP concentration for each sample was then obtained using CellTiter-Glo 2.0 (Promega) assay following the manufacturer’s protocol. Protein concentration for each sample was then determined using bicinchoninic acid assay (Thermo Fisher Scientific). ATP concentrations were then normalized to protein concentration for each sample. Statistical analysis was performed on five biological replicates.

### Seahorse Real-Time ATP Rate

shLuciferase or shBZW2 lentivirus transduced and pCDH/tag-free BZW2 transfected AsPC-1 and A549 cells were seeded (∼10^5^ cells) into a 96-well XF-96 cell culture plate (Agilent) and allowed to adhere overnight. Real-Time ATP Rate Assay (103592-100, Agilent) was performed according to manufacturer protocol using an Agilent's Seahorse Bioscience XF96 Extracellular Flux Analyzer. ATP production rates were determined using Wave analysis software (Agilent). Statistical analysis performed on five technical replicates for AsPC-1 cells and eight technical replicates for A549 cells.

### Seahorse Glycolytic Rate

shLuciferase or shBZW2 lentivirus transduced HT-29 and SW620 cells were seeded (∼10^5^ cells) into a 96-well XF-96 cell culture plate (Agilent) and allowed to adhere overnight. Glycolytic rate assay (103344-100, Agilent) was performed according to manufacturer protocol using an Agilent's Seahorse Bioscience XF96 Extracellular Flux Analyzer. Glycolytic rate was determined using Wave analysis software (Agilent). Statistical analysis was performed on seven technical replicates for HT-29 and SW620 cells. Differences were examined using an unpaired *t* test with Welch's correction. The *p*-values were indicated (∗*p* < 0.05 and ∗∗∗∗*p* < 0.0001).

### Calcium Measurement in Isolated Mitochondria

Stable shLuciferase or shBZW2 HEK293 cells were cultured in a 15-cm cell culture dish until ∼80% confluency and mitochondria was isolated using Qproteome Mitochondria Isolation Kit (Qiagen) following the manufacturer protocol. Lysate for cytosolic fraction was obtained in the process. Isolated mitochondria were then lysed with same lysis buffer from Qproteome kit, vortexed, and incubated on ice for 30 min. Lysates for both cytosolic and mitochondrial fractions were then diluted 10x with dH_2_O and calcium concentration was obtained for each sample using QuantiChrom Calcium Assay Kit (DICA-500) according to the manufacturer protocol (BioAssay Systems). Protein concentration of both cytosolic and mitochondrial fractions for each sample were then determined using BCA assay (Thermo Fisher Scientific). Calcium concentrations were normalized to protein concentration for each sample. Statistical analysis was performed on six biological replicates. Differences were examined using an unpaired *t* test with Welch's correction. The *p* values were indicated (∗∗∗*p* < 0.001 and ∗∗∗∗*p* < 0.0001).

### Western Blot Analysis of Isolated Mitochondria

Stable shLuciferase or shBZW2 HEK293 cells were cultured in a 15-cm cell culture dish until ∼80% confluency and mitochondria was isolated using Qproteome Mitochondria Isolation Kit (Qiagen) following the manufacturer protocol. Lysate for cytosolic fraction was obtained in the process. Isolated mitochondria were then lysed with same lysis buffer from Qproteome kit, vortexed, and incubated on ice for 30 min. Lysates for both cytosolic and mitochondrial fractions were analyzed using Western blot.

### MAM Isolation

Biochemical fractionation of HEK293 cells into pure cytosolic, ER (microsomal), mitochondrial, and mitochondrial-associated membrane (MAM) fractions was performed according to Karen Mossman (ed.), Innate Antiviral Immunity: Methods and Protocols, Methods in Molecular Biology, vol. 1656, p131-137 © Springer Science+Business Media LLC 2017 (28). Collected fractions were analyzed using Western blot.

### Anchorage-Independent Growth (Soft Agar) Assay

To each well of a 6-well plate, 2 ml of 0.6% base low-melting point agarose was added. After the agarose was solidified, 1.0 × 10^3^ of HCT116 cells transduced with five different BZW2 shRNAs or a control shLuciferase were mixed with 0.3% low-melting point agarose and plated into a 6-well plate on top of the 0.6% base agarose layer. Then 150 μl of normal culture medium was added on top of the 0.3% low-melting point agarose. The medium was changed every 48 h. After 14 days of culture, colonies were stained with 200 μl of Nitro Blue Tetrazolium Chloride staining solution (2 mg/ml in water, filtered) overnight at 37 °C. Plates were imaged using a Typhoon FLA 7000 scanner (GE Healthcare).

## Results

### Interactome Analysis Indicates that BZW2 Interacts with Both Mitochondrial and ER Proteins

To uncover potential functions of BZW2, we used a quantitative proteomic approach to identify its interacting proteins. We used a previously developed SILAC approach as it allows for more reliable identification of interacting proteins (24). Using this general approach, a Flag-tagged BZW2 was expressed in heavy-labeled cells, and an untagged BZW2 was equally expressed in light-labeled cells (supplemental Fig. S1*A*). Anti-Flag resin was then used to pull down BZW2 and its binding partners in both heavy- and light-labeled cells. The enriched samples were then mixed and subjected to LC-MS/MS analysis, and the identified proteins were quantified using the heavy and light labels. We expect that true interacting proteins would have higher heavy/light (H/L) ratios. This quantitative approach effectively removed false-positive proteins (*e.g.*, those that bind to the anti-Flag resin non-specifically) and thus allows reliable identification of true interacting proteins. To ensure reliability of the proteomics experiments, we carried out the SILAC studies in four replicates, including two forward SILAC experiments and two reverse SILAC experiments, and we only considered those proteins that were identified in at least three of the four replicates that meet our H/L ratio cut-off values as hits (see Experimental Procedures section for details).

**Fig. 1:**
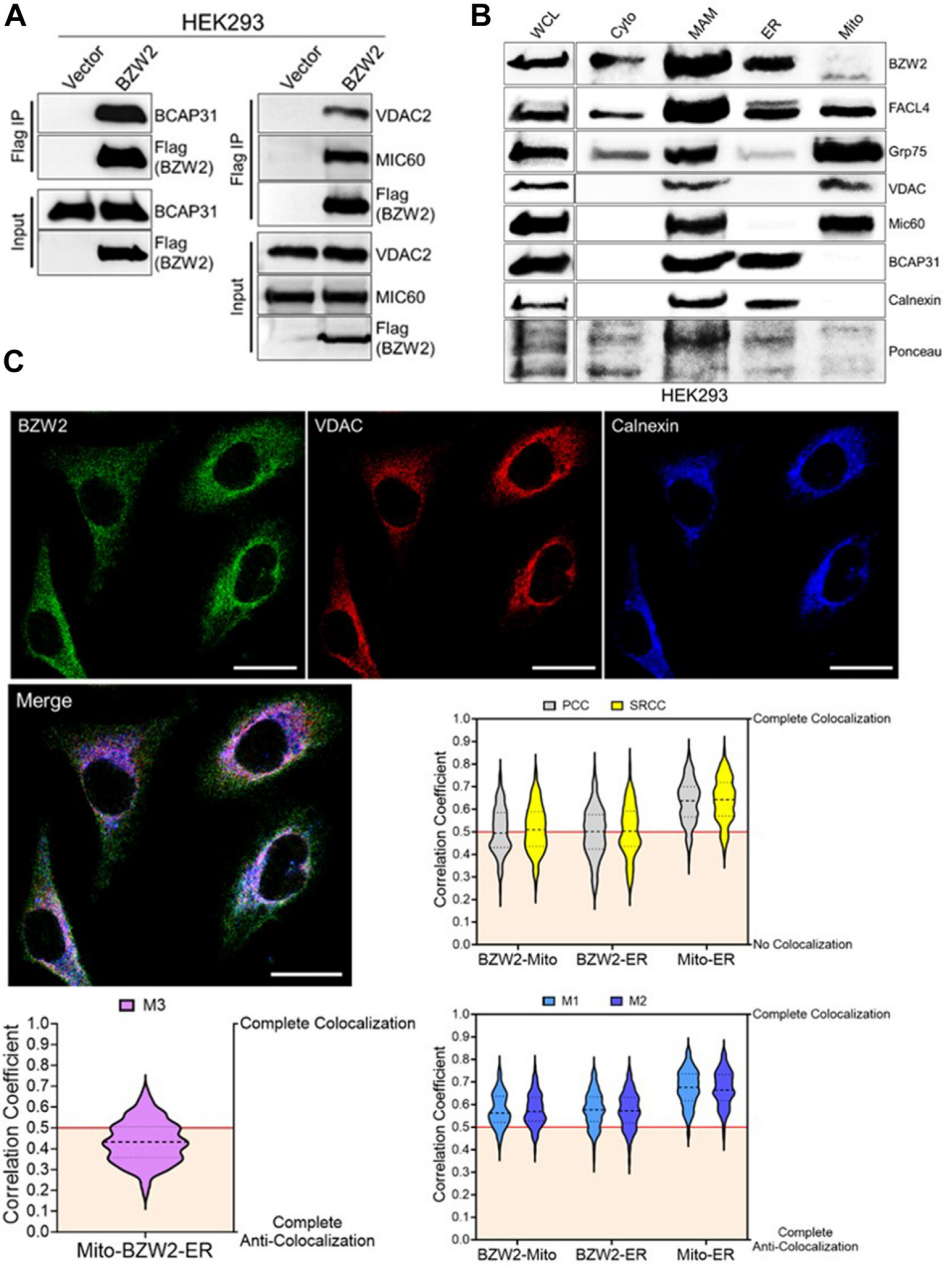
**BZW2 localizes to MERCS.***A*, immunoprecipitation of Flag-tagged BZW2 in HEK293 cells pulled down endogenous BCAP31, VDAC2, and MIC60. *B*, biochemical fractionation of HEK293 cells show BZW2 localization to ER, mitochondria, and mitochondria-associated membrane (MAM). *C*, representative confocal images showing endogenous BZW2, ER (calnexin), and mitochondria (VDAC) in AsPC-1 cells. Magnification: 63× oil; Scale bar represents 22 μm. Analysis of BZW2/ER/mitochondria colocalization using Pearson correlation coefficient (PCC) and Spearman’s rank correlation coefficient (SRCC) show BZW2 colocalization with both the ER and mitochondria. Extent of colocalization for PCC and SRCC coefficient values are defined: −1 = complete anti-colocalization; 0 = non-colocalization; 1 = complete colocalization. Analysis of BZW2/ER/mitochondria colocalization using Mander’s correlation coefficient (MCC, M1/M2 for two channel comparisons, M3 for three channels) show BZW2 colocalization with the ER, mitochondria, and MERCS. Extent of colocalization for MCC (M1, M2, M3) coefficient values are defined: 0 = complete anti-colocalization; 1 = complete colocalization (n = 121). BZW2, basic leucine zipper and W2 domain-containing protein 2; ER, endoplasmic reticulum; HEK, human embryonic kidney; MERCS, mitochondria-ER contact site.

Table 1 lists the names and reported cellular localization of interacting proteins identified for BZW2. To biochemically validate this, we focused on BCAP31 (ER membrane protein), VDAC2 (outer mitochondrial membrane (OMM) protein), and MIC60 (inner mitochondrial membrane (IMM) protein). Overexpression of Flag-tagged BZW2 in HEK293 cells followed by immunoprecipitation confirmed BZW2 interaction with BCAP31, VDAC2, and MIC60 (Fig. 1*A*).

**Table 1:** List of BZW2-interacting proteins identified by SILAC

Protein identified	Localization	Relevant reported functions
SLC25A5/ANT2	Mito	Ca^2^⁺ regulated mito ATP transporter/VDAC binding
SLC25A6/ANT3	Mito	Ca^2^⁺ regulated mito ATP transporter/VDAC binding
CHCHD3/Mic19	Mito	Cristae formation/ATP production
IMMT/Mic60	Mito	Cristae formation/ATP production
SLC25A3/PiC2	Mito	ATP production
ATP5A1	Mito	ATP production
SLC16A1/MCT1	Mito	Lactate importer
VDAC2	MERCS/Mito	Apoptosis/ATP, ADP, Ca^2^⁺ transport
AGK	Mito/ER	PTEN signaling/Lipid metabolism
PRDX1	Mito/Perox	PI3K signaling/Apoptosis
MUL1	Mito/Perox	Mito Ca^2^⁺
MBOAT7	ER	Lipid metabolism
CAMLG	ER	Lipid metabolism
SRPRB	ER	ER protein import
RPN2	ER	PI3K signaling
PGRMC1	Mito/ER	Ca^2^⁺ signaling
PGRMC2	ER/Cyto	Ca^2^⁺ signaling
BCAP31	MERCS/ER	Apoptosis/ER stress
VAPB	MERCS	Lipid metabolism
PTPLAD1/HACD3	ER/Mito/Perox	Lipid metabolism
TMEM43	Golgi/ER/Mito	Nucleus-mito contact
CKAP4	MERCS/ER/PM	ER-mito function/AKT signaling/VDAC binding
GNAI2	PM/Cyto/Vesicle	Cell proliferation
RALA	PM/Vesicle	PI3K-RAS signaling
BSG	PM/Vesicle	MCT importer
TMPO/LAP2	Nucleus	Cell cycle

From left to right: common name(s), reported cellular localization, relevant reported functions.

**Cyto:** cytosol, **Mito:** mitochondria, **Golgi:** golgi apparatus, **Perox:** peroxisome, **Vesicle:** cellular vesicle, **PM:** plasma membrane, **ER:** endoplasmic reticulum, **MERCS:** mitochondria-ER contact sites.

Our interactome results also identified an isoform-specific interaction with the small GTPase RalA. Because Ral (RAS-like) proteins can exist in either an active GTP-bound conformation or inactive GDP-bound conformation, we tested if the BZW2 interaction with RalA is nucleotide-dependent. Overexpression of both active and inactive mutants of RalA and RalB in HEK293 cells followed by immunoprecipitation showed that BZW2 interacts specifically with RalA and not RalB (supplemental Fig. S1*B*). No difference was observed in the extent of BZW2 interaction with the active or inactive mutants, suggesting that BZW2 is not a RalA effector protein. These biochemical data support the reliability of our interactome data.

In contrast to a previous report that BZW2 interacts with eIF2 and eIF3, these proteins were not identified in our interactome data. Other initiation and elongation factors, along with multiple 40S and 60S ribosomal subunits, were found in our results. However, their H/L ratios were outside the range of our cut off threshold (see Experimental Procedures section for details), and therefore our data do not support them being high-confidence BZW2-interacting proteins (supplemental Table S1).

### BZW2 Promotes Mitochondria-ER Contact Site Formation

While examining this interacting protein list, we noticed that BZW2 interacted with many mitochondrial and endoplasmic reticulum (ER) membrane proteins (Table 1). Interestingly, several proteins known to be involved in mitochondria-ER contact sites (MERCS) were also identified. This interactome data led us to hypothesize that BZW2 may be involved in MERCS formation and/or maintenance by interacting with both mitochondrial and ER membrane proteins.

To test this hypothesis, we examined whether the subcellular localization of endogenous BZW2 overlapped with MERCS. Using biochemical fractionation (28), we isolated the cytosolic, MAM, ER, and mitochondrial fractions in HEK293 cells. As expected, Western blot analysis showed high levels of endogenous BZW2 in the MAM fraction, comparable to MAM marker FACL4 (Fig. 1*B*). Interestingly, more BZW2 was observed in the pure ER fraction than the pure mitochondrial fraction.

According to Singh *et al.* 2011 (15), BZW2 contains an MA3 and W2 domain. We wondered whether different domains of BZW2 may be responsible for its specific organelle localization. To investigate this, we generated a Flag-tagged BZW2 construct with a W2 domain deletion (BZW2ΔW2) as well as the W2 domain of BZW2 alone (BZW2-W2) and compared the interactions with the full-length protein (BZW2-FL). HEK293 cells were transfected with these constructs followed by Flag IP and Western blot analysis (supplemental Fig. S2*B*). The results show that the deletion of the W2 domain prevented BZW2 interaction with the ER protein BCAP31 but increased its interaction with both the IMM protein Mic60 and OMM protein VDAC2. Interestingly, the BZW2-W2 protein had increased interaction with the OMM protein VDAC2 and ER protein BCAP31 but had decreased interaction with IMM protein Mic60. These findings suggest that the W2 domain of BZW2 can interact with both ER and OMM proteins and likely mediates its effect in promoting MERCS, while the rest of BZW2 promotes its interaction with IMM proteins.

Additionally, we used confocal microscopy to measure the colocalization between endogenous BZW2 and markers of the ER (calnexin immunolabeling) and/or mitochondria (VDAC immunolabeling). For two-channel comparisons (*i.e.*, BZW2-ER, BZW2-mito, ER-mito) (supplemental Fig. S2*A*), colocalization was analyzed using PCC, SRCC, and Mander’s correlation coefficients (M1 and M2). For three-channel comparisons (*i.e.*, BZW2-ER-mito), colocalization was analyzed using Mander’s correlation coefficient M3 (Fig. 1*C*) (27). The coefficient values for PCC, SRCC, M1, and M2 revealed substantial colocalization of endogenous BZW2 with both the ER and mitochondrial markers in AsPC-1 cells. Further, the M3 coefficient values revealed substantial BZW2/ER/mitochondria colocalization. Together, these data support BZW2 localization to MERCS.

**Fig. 2:**
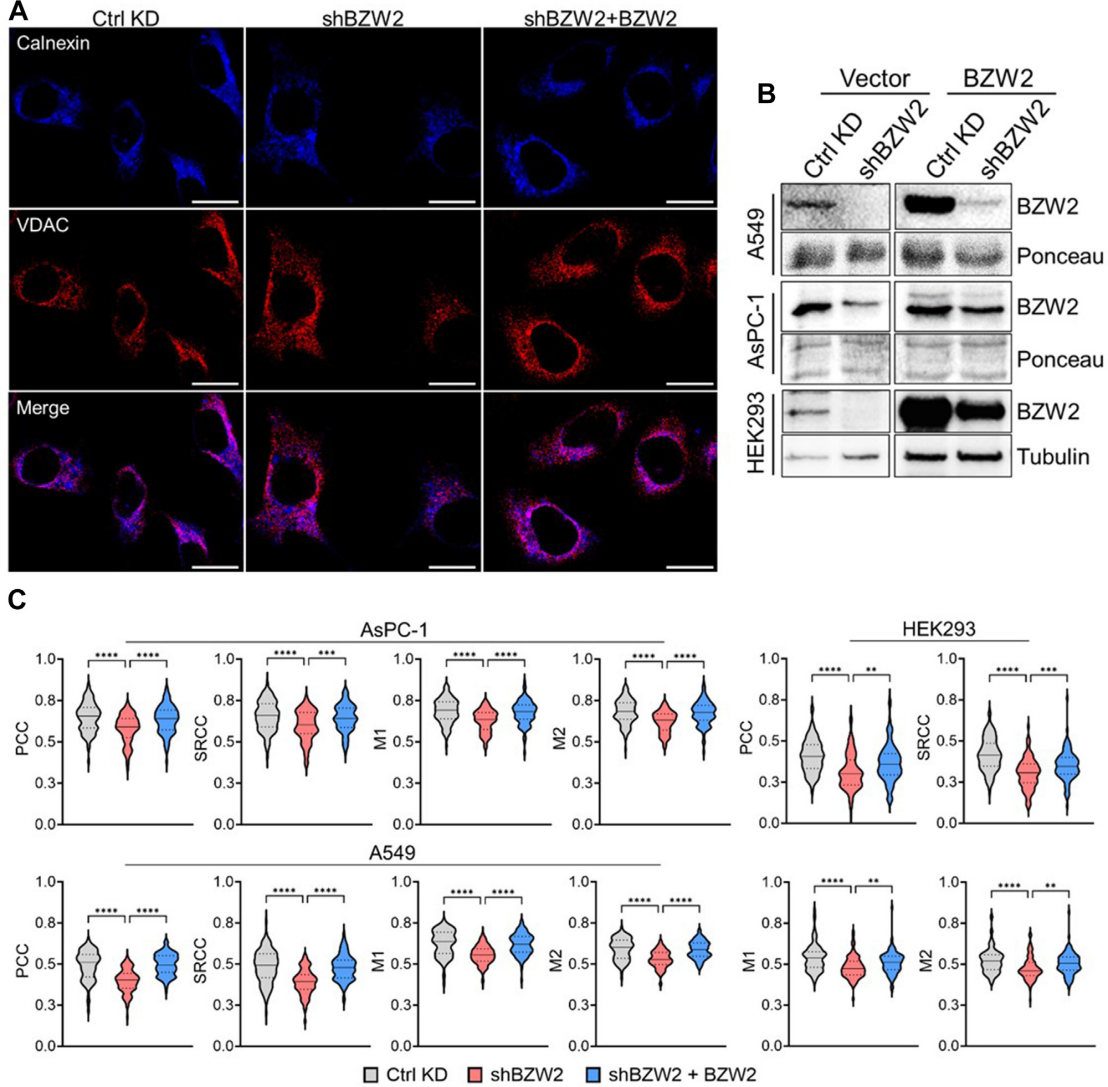
**BZW2 promotes mitochondria-ER contact.***A*, representative confocal images showing the colocalization of ER (Calnexin) and mitochondria (VDAC) in AsPC-1 cells with transient BZW2 KD and overexpression. Magnification: 63× oil; Scale bar represents 22 μm. *B*, Western blot showing transient BZW2 KD and re-expression in AsPC-1, A549, and HEK293 cells. *C*, statistical analyses of colocalization using PCC, SRCC, and MCC shows a decrease in ER-mito contact when BZW2 is knocked down and an increase in ER-mito contact when BZW2 is re-introduced in AsPC-1, A549, and HEK293 cells (n ≥ 100). ∗∗*p* < 0.01, ∗∗∗*p* < 0.001 and ∗∗∗∗*p* < 0.0001. BZW2, basic leucine zipper and W2 domain-containing protein 2; ER, endoplasmic reticulum; HEK, human embryonic kidney; KD, knockdown; PCC, Pearson correlation coefficient; SRCC, Spearman’s rank correlation coefficient.

To assess if BZW2 promotes MERCS, we knocked down BZW2 levels using shRNA and used confocal microscopy to measure ER-mitochondria colocalization. ER and mitochondria were marked using Calnexin and VDAC antibodies, respectively. To verify that an observed phenotype was not cell line–dependent, we performed the BZW2 KD experiments in As-PC1, A549, and HEK293 cells. To verify that an effect was on-target, we re-expressed BZW2 in the KD cells to rescue the KD-induced phenotypes (Fig. 2, *A* and *B*, and supplemental Fig. S3). Colocalization analysis using PCC, SRCC, M1, and M2 showed that BZW2 KD decreased the extent of ER-mitochondria colocalization in As-PC1, A549, and HEK293 cells. Re-expression of BZW2 in the KD cells restored ER-mitochondria colocalization, showing that BZW2 promotes MERCS (Fig. 2*C*).

### BZW2 Promotes Mitochondrial Calcium Levels

**Fig. 3:**
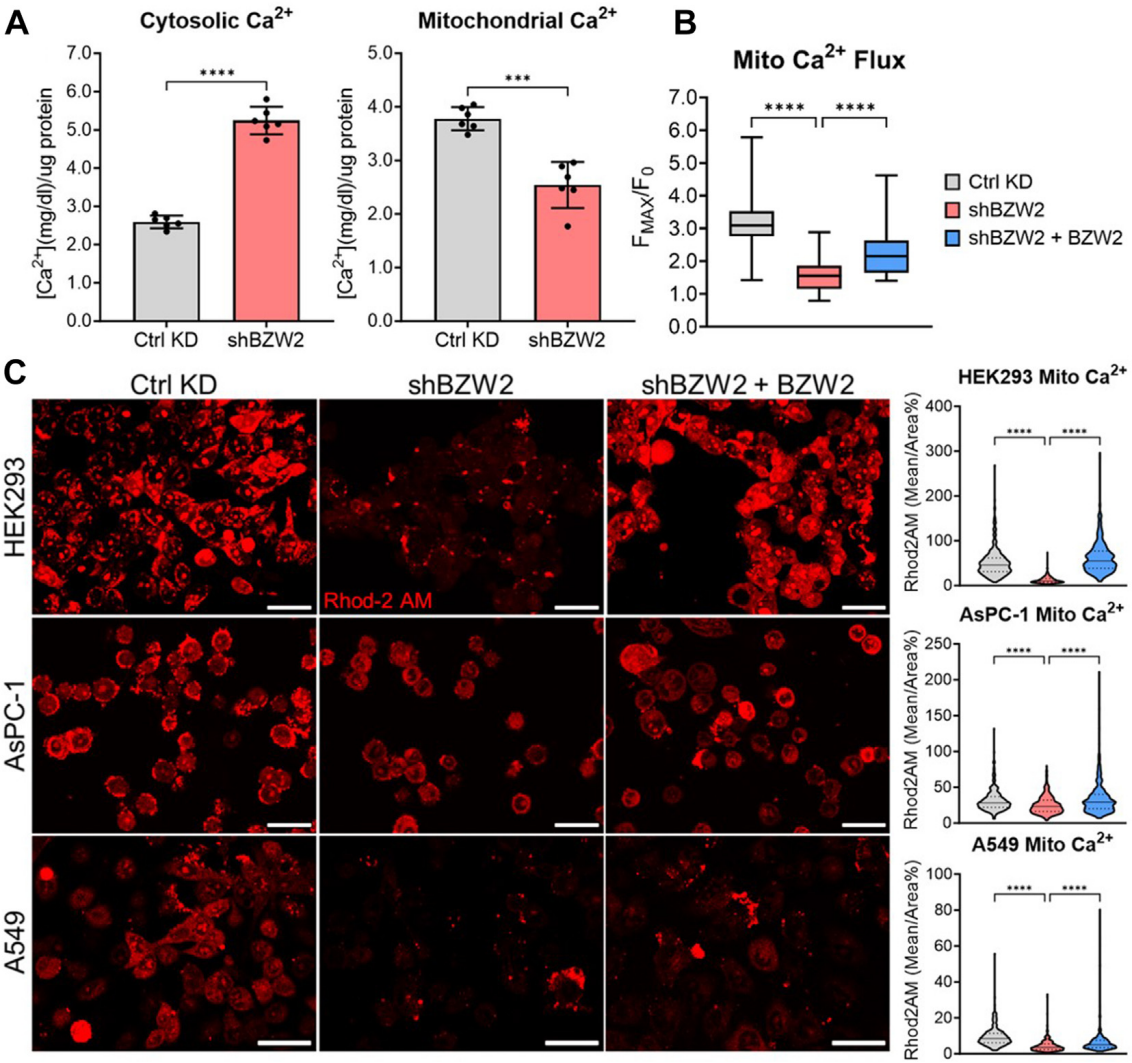
**BZW2 promotes mitochondria calcium uptake.***A*, analysis of isolated mitochondria and cytosolic calcium levels show an increase in cytosolic calcium but a decrease in mitochondrial calcium levels when BZW2 is stably knocked down in HEK293 cells (n = 6). ∗∗∗*p* < 0.001 and ∗∗∗∗*p* < 0.0001. *B*, analysis of mitochondrial calcium flux using Rhod-2 AM in live HEK293 cells with transient BZW2 KD and re-expression shows that BZW2 promotes calcium flux into the mitochondria. ∗∗∗∗*p* < 0.0001. *C*, representative confocal images of mitochondrial calcium levels using Rhod-2 AM in live HEK293, AsPC-1, and A549 cells with transient BZW2 KD and re-expression. Magnification: 40× water; Scale bar represents 22 μm. Statistical analyses of live cell mitochondrial calcium show a decrease in mitochondrial calcium levels when BZW2 is knocked down and an increase when BZW2 is re-introduced in HEK293, AsPC-1, and A549 cells (n ≥ 300). ∗∗∗∗*p* < 0.0001. BZW2, basic leucine zipper and W2 domain-containing protein 2; HEK, human embryonic kidney; KD, knockdown.

A key function of mitochondria-ER contact sites is to promote the transfer of calcium from the ER to the mitochondria (29, 30, 31, 32, 33, 34, 35, 36, 37). Considering that BZW2 promotes mitochondria-ER contact and interacts with the mitochondrial calcium importer VDAC2, we hypothesized that BZW2 might regulate mitochondrial calcium levels. We isolated mitochondria from HEK293 cells and measured the calcium concentrations in both the cytosolic fraction and isolated mitochondria directly (Fig. 3*A*). We observed an increase in cytosolic calcium and a decrease in isolated mitochondria calcium concentration upon BZW2 KD.

To measure changes in mitochondrial calcium levels in live cells, we used the mitochondrial calcium probe Rhod-2 AM. HEK293 cells (control KD, BZW2 KD, or BZW2 KD and with BZW2 re-expression) were loaded with Rhod-2 AM and the mitochondrial calcium flux was measured using confocal imaging (Fig. 3*B* and supplemental Fig. S4*B*). BZW2 KD dramatically decreased, while re-expression of BZW2 restored mitochondrial calcium flux, supporting that BZW2 indeed promotes mitochondrial calcium uptake. To ensure the observed phenotype was specific to mitochondrial calcium, we also measured the calcium flux using the reduced form of Rhod-2 AM (which has been shown to increase mitochondrial specificity (38)) and the mitochondrial calcium florescent protein Mito4x-GCaMP6f (39). We observed the same changes in mitochondrial calcium flux using both the reduced Rhod-2AM and Mito4x-GCaMP6f, confirming the reliability of Rhod-2 AM measurements (supplemental Fig. S4*C*). Finally, to validate the lower levels of mitochondrial calcium observed in the isolated mitochondria of HEK293 BZW2 KD cells, we measured the basal level of mitochondrial calcium using Rhod-2 AM in HEK293, AsPC-1, and A549 cells with BZW2 KD or BZW2 KD and re-expression. As expected, BZW2 KD decreased basal mitochondrial calcium levels while re-expression of BZW2 recovered basal levels in all three cell lines (Fig. 3*C*).

### BZW2 Promotes ATP Production

**Fig. 4:**
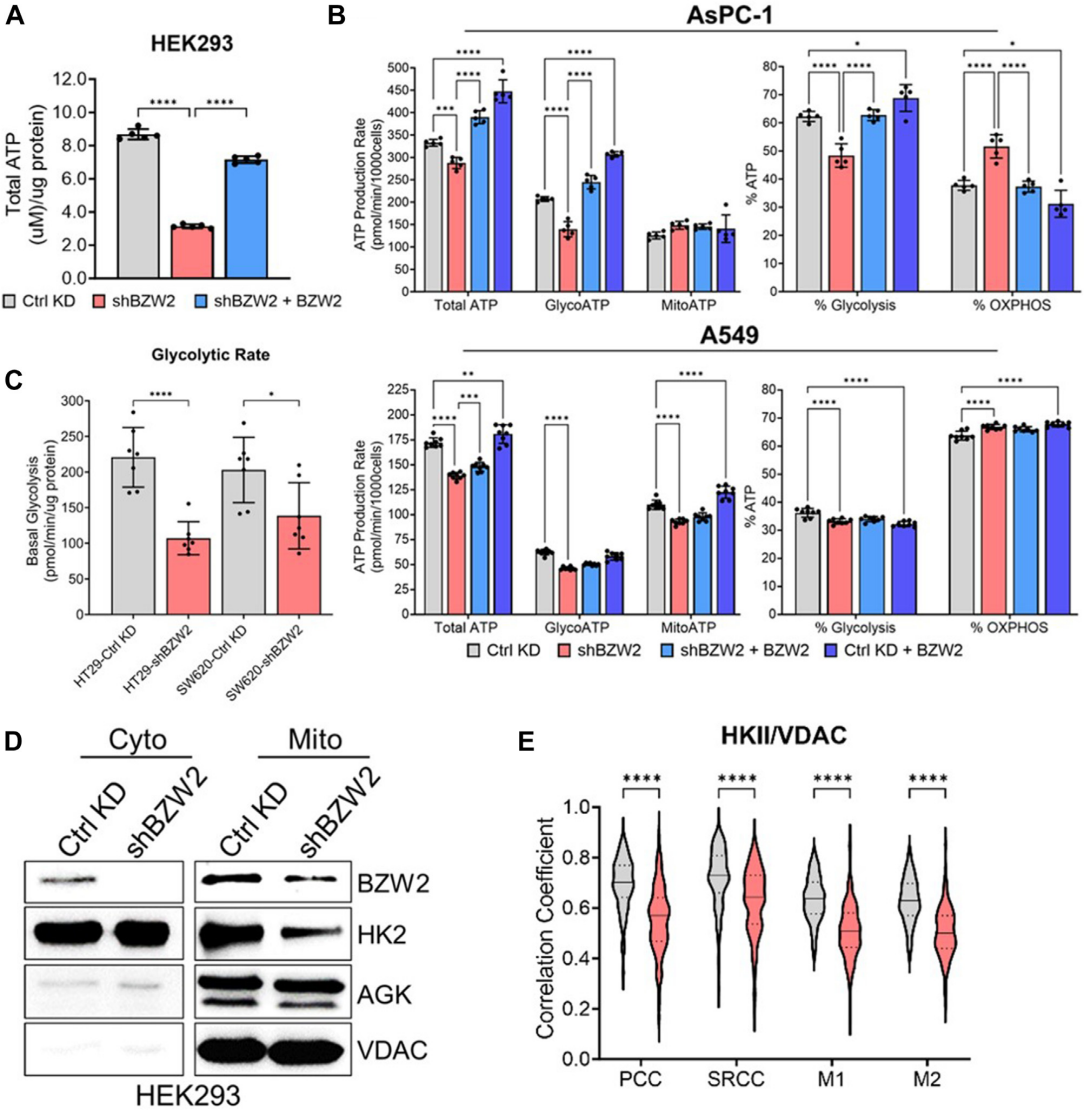
**BZW2 promotes ATP generation.***A*, analyses of total ATP levels show a decrease in total ATP when BZW2 is knocked down and an increase when BZW2 is re-introduced in HEK293 cells (n = 5). ∗∗∗∗*p* < 0.0001. *B*, analyses of total, glycolytic, and mitochondrial ATP production, as well as the relative percentage ATP production, when BZW2 is knocked down and expressed in both control KD and BZW2 KD AsPC-1 cells (*top panel*). (n = 5) ∗*p* < 0.05, ∗∗∗*p* < 0.001, and ∗∗∗∗*p* < 0.0001. Analyses of total, glycolytic, and mitochondrial ATP production, as well as the relative percentage ATP production, when BZW2 is knocked down and expressed in both control KD and BZW2 KD A549 cells (*bottom panel*). (n = 8) ∗∗*p* < 0.01, ∗∗∗*p* < 0.001, and ∗∗∗∗*p* < 0.0001. *C*, statistical analysis of glycolytic rate shows a decrease in glycolytic rate when BZW2 is knocked down in HT-29 and SW620 cells (n = 7). ∗*p* < 0.05, ∗∗∗∗*p* < 0.00 01. *D*, Western blot analysis of isolated mitochondrial and cytosolic fractions of HEK293 cells shows a significant decrease in HKII localization to mitochondria when BZW2 is knocked down. AGK is shown as an additional mitochondrial protein reference. *E*, analyses of HKII/VDAC colocalization using PCC, SRCC, and MCC show a decrease in HKII localization to the mitochondria when BZW2 is knocked down in HEK293 cells (n ≥ 135). ∗∗∗∗*p* < 0.0001. BZW2, basic leucine zipper and W2 domain-containing protein 2; HEK, human embryonic kidney; HKII, hexokinase II; KD, knockdown; PCC, Pearson correlation coefficient; SRCC, Spearman’s rank correlation coefficient.

Mitochondrial calcium pools activate several TCA cycle enzymes and thus promote mitochondrial metabolism and ATP production (35, 37, 40, 41). Therefore, we decided to test if BZW2 KD affects cellular ATP levels. As expected, BZW2 KD led to a decrease in total ATP, whereas reintroduction of BZW2 recovered total cellular ATP levels in HEK293 cells (Fig. 4*A*).

Next, we wanted to distinguish between ATP generated from oxidative phosphorylation (OXPHOS) in mitochondria compared to glycolysis. To test this, we measured ATP production using the Seahorse real-time ATP assay (Agilent) in AsPC-1 and A549 cells. The results again showed that BZW2 KD decreased total ATP production and reintroduction of BZW2 restored total ATP production in both cell lines (Fig. 4*B*). Analyzing the glycolytic and mitochondrial ATP production, we found that BZW2 primarily promotes glycolytic ATP production in AsPC-1 cells while promoting both glycolytic and mitochondrial ATP production in A549 cells. In AsPC-1 cells, glycolytic ATP production was decreased when BZW2 was knocked down and increased when BZW2 is expressed in both control KD and BZW2 KD cells. Conversely, AsPC-1 mitochondrial ATP was not significantly affected by BZW2, although there was a slight increase when BZW2 was knocked down. In A549 cells, both glycolytic and mitochondrial ATP production were decreased when BZW2 was knocked down. Interestingly, when examining the percentage of glycolytic and mitochondrial ATP production in both cell lines, BZW2 KD decreased the glycolytic ATP percentage while increasing the OXPHOS ATP percentage.

To test if BZW2 KD inhibits glycolysis in other cell lines, we knocked down BZW2 in HT-29 and SW620 cells and performed a Seahorse glycolytic rate assay (Agilent) (Figs. 4*C* and supplemental Fig. S5*A*). Knocking down BZW2 decreased the glycolytic rate in HT-29 and SW620 cells, consistent with the result in AsPC-1 and A549 cells.

It is known that pyruvate dehydrogenase can be activated by calcium (42). Thus, BZW2 KD can decrease mitochondrial calcium and thus, inhibit pyruvate dehydrogenase, which in turn will slow down glycolysis. A glycolysis enzyme, hexokinase II (HKII), is known to interact with VDAC linking glycolysis to mitochondrial OXPHOS by allowing HKII access to mitochondrial ATP (43). Therefore, we hypothesized that HKII mitochondrial localization might decrease when BZW2 is knocked down. To test this, we biochemically fractionated control KD and BZW2 KD HEK293 cells into mitochondria and cytosolic fractions and visualized endogenous HKII and VDAC *via* Western blot (Fig. 4*D* and supplemental Fig. S5*B*). AGK levels were used as an additional mitochondrial control. As expected, HKII mitochondrial localization was drastically decreased in the BZW2 KD cells. To test if HKII interaction with VDAC decreases, we measured endogenous VDAC and HKII colocalization in HEK293 cells using immunolabeling and confocal microscopy (Fig. 4*E* and supplemental Fig. S5*C*). The coefficient values PCC, SRCC, M1, and M2 for HKII-VDAC colocalization decreased substantially when BZW2 was knocked down. These data are consistent with the decrease of glycolytic ATP production when BZW2 was knocked down and further support that BZW2 promotes glycolysis and glycolytic ATP production by promoting mitochondrial metabolism.

### BZW2 Promotes TAG/LD Levels and Inhibits AMPK-Induced Fatty Acid Oxidation

**Fig. 5:**
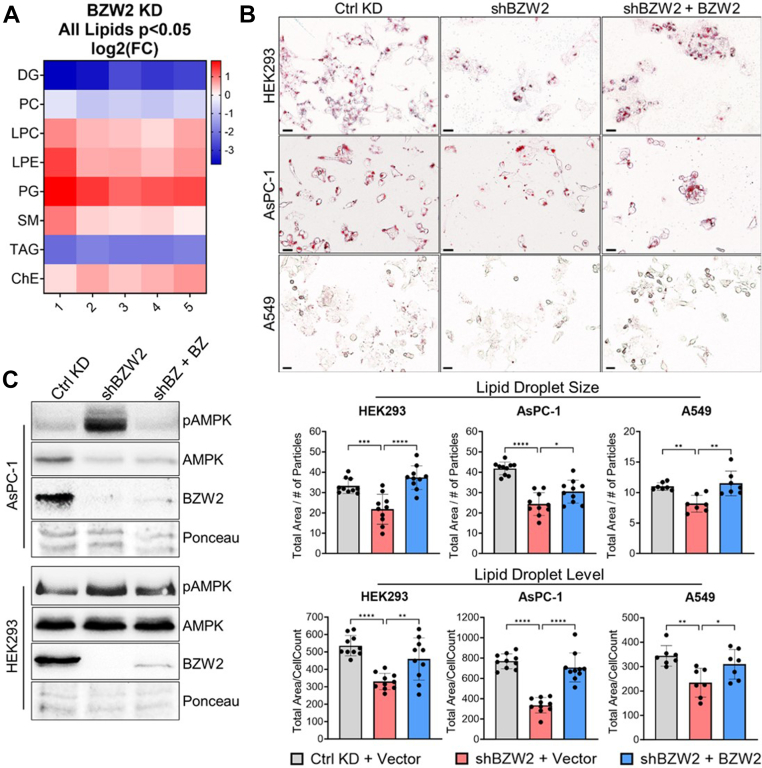
**BZW2 promotes TAG/lipid droplet levels and regulates APMK phosphorylation.***A*, heatmap of the log2 of the fold change (FC) in total abundance for each lipid class with a *p*-value <0.05 in HEK293 cells shows a decrease in triacylglycerol (TAG) levels when BZW2 is stably knocked down (n = 5). *B*, representative Cytation 5 images of LD levels using Oil Red O in HEK293, AsPC-1, and A549 cells with transient BZW2 KD and re-expression. Magnification: 20× color brightfield. Scale bar represents 22 μm. Statistical analyses (*bottom panels*) of LD size and LD levels show a decrease in both LD size and level when BZW2 is knocked down and an increase when BZW2 is reintroduced (HEK293 and AsPC-1: n = 10, A549: n = 7; total cells/sample in each cell line ≥120). ∗*p* < 0.05, ∗∗*p* < 0.01, ∗∗∗*p* < 0.001, and ∗∗∗∗*p* < 0.0001. *C*, Western blot showing an increase in AMPK phosphorylation (T172) when BZW2 is knocked down and a return to normal levels when BZW2 is reintroduced in AsPC-1 and HEK293 cells. BZW2, basic leucine zipper and W2 domain-containing protein 2; HEK, human embryonic kidney; KD, knockdown; LD, lipid droplet.

MERCS also play key roles in lipid metabolism, including phospholipid biosynthesis and exchange between the ER and mitochondria (34, 44, 45, 46, 47, 48). Because BZW2 promotes MERCS, we hypothesized that BZW2 KD would also affect lipid metabolism. Therefore, we performed a lipidomics analysis in BZW2 KD compared to control HEK293 cells. This analysis identified 770 unique lipid species, which were grouped into their respective lipid class. The total abundance of each lipid class was then combined, allowing for a statistical analysis of both control and BZW2 KD cells. The changes in abundance (plotted as log2) for all groups exhibiting statistical significance (*p* < 0.05) were then compared using a heatmap (Fig. 5*A* and supplemental Fig. S6).

The most prominent change in lipid composition when knocking down BZW2 was the downregulation of triacylglycerol (TAG) levels. Because TAG is stored in LD (45, 48), we visualized LD formation using Oil Red O staining in HEK293, AsPC-1, and A549 cells. Consistent with the lipidomics results, LD size and overall levels decreased upon BZW2 KD, and these phenotypes were restored when BZW2 was re-expressed (Fig. 5*B*).

The observed decrease in TAG/LD levels in BZW2 KD cells could be due to a decrease in lipid synthesis or an increase in fatty acid oxidation (FAO). Given the previously observed higher cytosolic calcium levels (Fig. 3*A*) and lower total cellular ATP levels (Fig. 4*A*), we suspected that BZW2 KD might activate AMPK, which would promote FAO (35, 41, 45, 49, 50, 51). We therefore assessed AMPK activation by probing for the phosphorylation of T172 of AMPK *via* Western blot. We indeed observed an increase of AMPK phosphorylation in BZW2 KD HEK293, AsPC-1, and A549 cells, which was rescued by re-expression of BZW2 (Fig. 5*C* and supplemental Fig. S6*C*). These results suggest that the decrease in TAG/LD levels in BZW2 KD cells were likely due to AMPK-induced FAO. The activation of AMPK and FAO may also explain our finding that BZW2 KD decreased glycolytic ATP production but had little or opposite effect on mitochondrial ATP production, as FAO can compensate mitochondrial ATP production.

## Discussion

**Fig. 6:**
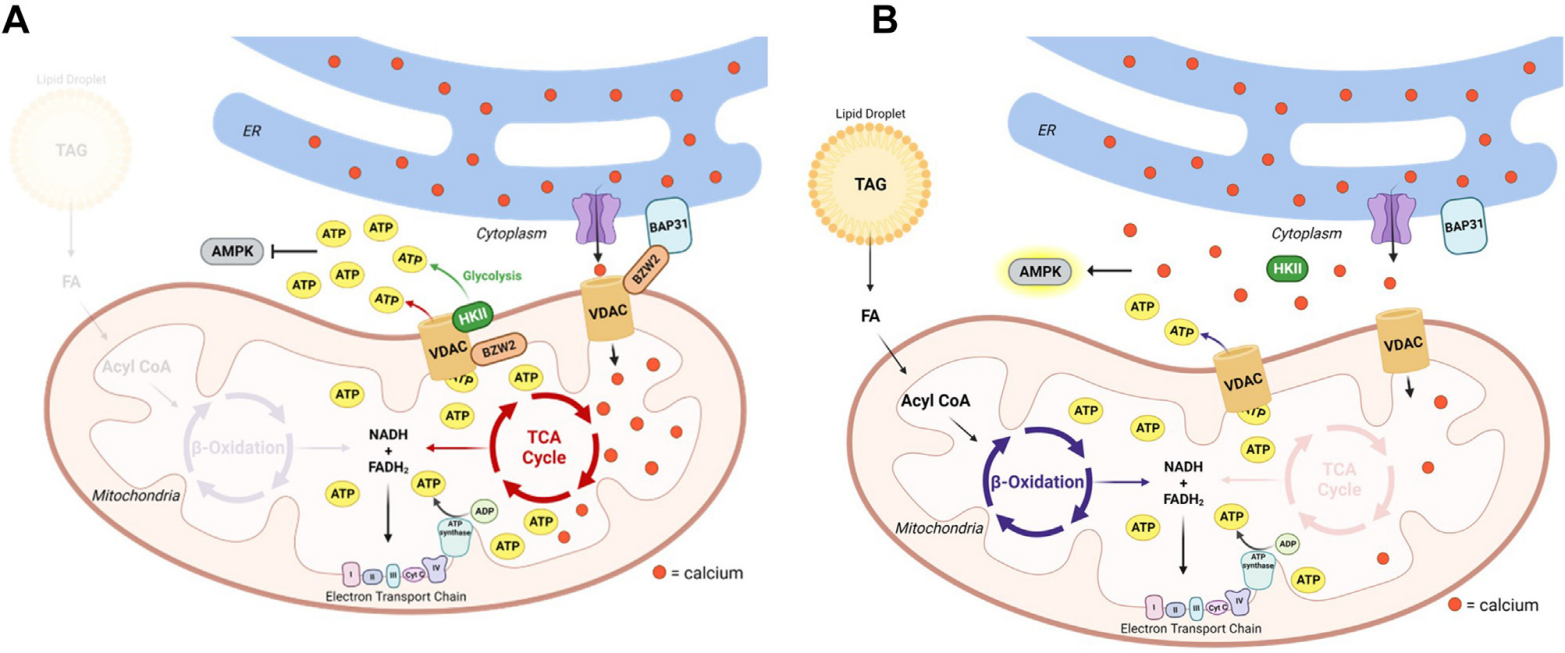
**A model for the proposed BZW2 molecular function.***A*, BZW2 promotes calcium flow from the ER into the mitochondria by promoting MERCS. Increased mitochondrial calcium promotes TCA cycle ATP generation, HKII mitochondrial localization, and glycolytic ATP production. High ATP and low Ca^2+^ levels in the cytosol inhibit AMPK-induced fatty acid oxidation (FAO). *B*, knocking down BZW2 results in a decrease in MERCS, mitochondrial Ca^2+^ levels, and HKII mitochondrial localization. Lower ATP and higher Ca^2+^ levels in the cytosol activate AMPK and promote FAO, which compensates for decreased TCA cycle–driven ATP production. BZW2, basic leucine zipper and W2 domain-containing protein 2; ER, endoplasmic reticulum; HKII, hexokinase II; MERCS, mitochondria-ER contact site.

Using a quantitative interactome approach, we discovered previously unknown molecular functions for a lesser studied protein, BZW2. We found that BZW2 promotes MERCS by interacting with both ER and mitochondrial proteins. By enhancing MERCS, BZW2 facilitates calcium entry from the ER into the mitochondria which activates TCA cycle enzymes and maintains mitochondrial ATP production. BZW2 maintains glycolytic ATP production by promoting pyruvate (a glycolysis end product) metabolism and the mitochondrial localization of HKII. Consequently, maintaining MERCS and HKII-induced glycolysis keeps cytosolic ATP levels high, preventing APMK activation and slowing down fatty acid oxidation (Fig. 6*A*).

While this model explains most of the effects we observed when BZW2 is knocked down, the increased percentage of ATP produced from mitochondria when BZW2 is knocked down was initially puzzling. A potential explanation is that FAO is compensating for TCA cycle inhibition. When BZW2 is knocked down, an increase in APMK phosphorylation and a decrease in lipid droplet/TAG levels were also observed, which strongly implies that β-oxidation is taking place. Though lower mitochondrial calcium levels inhibit several TCA cycle enzymes, the FADH_2_/NADH produced from the β-oxidation of fatty acids can feed directly into the electron transport chain and compensate for the reduced mitochondrial ATP production (Fig. 6). The increase in mitochondrial ATP production when knocking down BZW2 was more pronounced in AsPC1 cells. A possible explanation for this could be that AsPC-1 has significantly higher levels of energy stored in lipid droplets than A549. In Figure 5*B*, we noticed that AsPC-1 had ∼2.5× higher lipid droplet levels and ∼4× larger lipid droplet size than A549. Thus, it is likely that AsPC-1 had more TAG to oxidize and can produce more ATP through FAO when needed, which could explain why mitochondrial ATP production was not decreased by BZW2 KD in AsPC-1 cells.

Our model raises an interesting question of how BZW2 interacts with proteins located in separate cellular compartments or on different organelle membranes. For instance, in Figure 1*A*, immunoprecipitation shows that BZW2 interacts with the ER transmembrane protein BCAP31 as well as the OMM protein VDAC2 and IMM protein Mic60. Regarding BZW2 interaction with the IMM, Mic60 has been shown to bridge the IMM and OMM by associating with several OMM proteins involved in mitochondrial protein import and assembly, including the translocase of the outer membrane protein, the sorting and assembly machinery, and VDAC (52). Therefore, it is possible that BZW2 interacts with Mic60 indirectly through OMM interactions. Our data indicates that the W2 domain of BZW2 alone shows a drastic increase in interaction with both the OMM and ER, yet a significantly weaker interaction with the IMM (supplemental Fig. S2*B*). This indicates that BZW2 probably does not interact with Mic60 indirectly through VDAC2. Perhaps a particular motif in either the MA3 domain or the N-terminal sequence of BZW2 allows for translocation into the mitochondrial intermembrane space, allowing it to interact with Mic60.

Our data shows that the deletion of the W2 domain of BZW2 abolishes interaction with the ER membrane (supplemental Fig. S2*B*) and the W2 domain alone is enough to interact with both ER protein BCAP31 and OMM protein VDAC2. This suggests that the W2 domain of BZW2 is likely the tether that connects ER and OMM proteins to promote MERCS.

BZW2 has been reported to affect translation by directly binding to eIF2 and eIF3, competing with eIF2-eIF5 complex formation, and thus altering the timing of translation initiation resulting in translation of alternative ORFs (2, 11, 12, 13, 14, 15). In contrast to this proposed function, our interactome analysis did not identify eIF2 or eIF3 as strong BZW2 interaction partners. This discrepancy could be due to several reasons. First, previous reports of BZW2 regulating translation through direct interaction with eIF2 were predominantly limited to *in vitro* experiments or studies of their yeast orthologs. It is possible that the BZW2 interaction with eIF2 and eIF3 is weaker in mammalian cells. Second, previous proteomics results in HEK293T cells to identify BZW2-interacting proteins were not performed in a quantitative manner; in those studies, the affinity beads used to pull down BZW2 were washed minimally to maintain the protein complex (11). This protocol likely resulted in identification of some nonspecifically bound proteins. This highlights the importance of the reliability of our SILAC quantification and experimental method. The use of Flag-tagged BZW2 with no-tag BZW2 as a negative control helps to remove many nonspecific factors.

Considering our biochemical fractionation data indicates BZW2 primarily localizes to the ER membrane, where pools of ribosomes are enriched, it is possible that initiation factors and ribosomal proteins were pulled down due to the subcellular localization. A close examination of our raw proteomics data revealed several initiation factors and 40S and 60S ribosomal subunits; however, their enrichment, quantified as H/L ratios, were modest and outside the range of our cut-off threshold. Furthermore, our finding that BZW2 promotes ATP production could potentially explain reports of BZW2 regulating start codon selection because ATP hydrolysis regulates multiple steps in translation, including translation initiation (53, 54, 55, 56).

Previous reports also indicated that BZW2 KD decreased cell proliferation, AKT signaling, ERK activation, and increased apoptosis, although the molecular mechanisms underlying these effects are unknown. Our finding that BZW2 promotes MERCS, mitochondrial metabolism, and glycolysis could potentially explain these reported effects. The decreased AKT signaling, ERK activation, and cell proliferation caused by BZW2 KD are consistent with the role of BZW2 in promoting ATP production. Regarding apoptosis, VDAC2 interacts with Bak (Bcl-2 associated killer protein) and inhibits apoptosis (33, 57, 58). Our data supports a BZW2–VDAC2 interaction, which may in turn affect the VDAC2 interaction with Bak and thus apoptosis. Additionally, HKII detachment from the mitochondria has been shown to induce apoptosis (59). This model may explain observations that BZW2 KD increases apoptosis.

While our interaction data did not completely agree with previous publications, we did also observe a decrease in AKT signaling and c-Myc expression levels upon BZW2 KD (suppleemntal Fig. S7*A*), in agreement with several earlier reports (2, 4, 5, 8, 16). We also observed an inhibition of anchorage-independent growth upon BZW2 KD (suppleemntal Fig. S7*B*), consistent with multiple reports roles for BZW2 in tumorigenesis. Finally, further supporting our proteomics results, three proteins identified in our interactome (PTPLAD1, TMEM43, MUL1) were independently verified as BZW2 interactors in high-throughput studies cataloged on the IntAct and BioGRID databases (60, 61, 62).

Recently, it has been increasingly recognized that most research attention is given to proteins that already have well-known functions, and there is an urgent need to elucidate functional information for the lesser studied proteins. Our study not only reveals important insights into the molecular function of BZW2, but it also provides an example of how high-quality interactome data can be harnessed to understand the function of an understudied protein. In the BZW2 case, the interactome data allowed us to connect BZW2 to MERCS. Previously, we also reported an example that interactome data allows us to identify the enzyme that reduces an iron-containing protein (23). Conversely, we also reported an example that interactome data enables the identification of substrate proteins for N-terminal glycine myristoyltransferase (22). These examples together highlight different ways that interactome data can serve valuable hypothesis-generating roles that enable mechanistic understandings of the molecular functions of understudied proteins.

## Data Availability

The mass spectrometry proteomics data have been deposited to the ProteomeXchange Consortium *via* the PRIDE (63) partner repository with the dataset identifier PXD041354.

## Supplemental Data

This article contains supplemental data.

## Conflict of interest

H. L. is a founder and consultant for Sedec Therapeutics.

## References

[1] Kustatscher G., Collins T., Gingras A.C., Guo T., Hermjakob H., Ideker T. (2022). Understudied proteins: opportunities and challenges for functional proteomics. Nat. Methods.

[2] Sato K., Masuda T., Hu Q., Tobo T., Gillaspie S., Niida A. (2019). Novel oncogene 5MP1 reprograms c-Myc translation initiation to drive malignant phenotypes in colorectal cancer. EBioMedicine.

[3] Liu J., Yang T., Zhang Y., Wang S. (2020). Promotion of BZW2 by LINC00174 through miR-4500 inhibition enhances proliferation and apoptosis evasion in laryngeal papilloma. Cancer Cell Int..

[4] Cheng D.-D., Li S.J., Zhu B., Yuan T., Yang Q.C., Fan C.Y. (2017). Downregulation of BZW2 inhibits osteosarcoma cell growth by inactivating the Akt/mTOR signaling pathway. Oncol. Rep..

[5] Li G., Lu A., Chen A., Geng S., Xu Y., Chen X. (2021). BZW2/5MP1 acts as a promising target in hepatocellular carcinoma. J. Cancer.

[6] Gao H., Yu G., Zhang X., Yu S., Sun Y., Li Y. (2019). BZW2 gene knockdown induces cell growth inhibition, G1 arrest and apoptosis in muscle-invasive bladder cancers: a microarray pathway analysis. J. Cell. Mol. Med..

[7] Nachmias B., Khan D.H., Voisin V., Mer A.S., Thomas G.E., Segev N. (2022). IPO11 regulates the nuclear import of BZW1/2 and is necessary for AML cells and stem cells. Leukemia.

[8] Jin X., Liao M., Zhang L., Yang M., Zhao J. (2019). Role of the novel gene BZW2 in the development of hepatocellular carcinoma. J. Cell. Physiol..

[9] Liu L., Zhao J., Peng Y., Yang M., Zhang L., Jin X. (2020). miR-let-7a-5p inhibits invasion and migration of hepatoma cells by regulating BZW2 expression. Onco Targets Ther..

[10] Long X., Li J., Wen F., Cao Y., Luo Z., Luo C. (2022). miR-140-3p attenuated the tumorigenesis of multiple myeloma *via* attenuating BZW2. Hematology.

[11] Singh C.R., Glineburg M.R., Moore C., Tani N., Jaiswal R., Zou Y. (2021). Human oncoprotein 5MP suppresses general and repeat-associated non-AUG translation *via* eIF3 by a common mechanism. Cell Rep..

[12] Loughran G., Firth A.E., Atkins J.F., Ivanov I.P. (2018). Translational autoregulation of BZW1 and BZW2 expression by modulating the stringency of start codon selection. PLoS One.

[13] Hiraishi H., Oatman J., Haller S.L., Blunk L., McGivern B., Morris J. (2014). Essential role of eIF5-mimic protein in animal development is linked to control of ATF4 expression. Nucleic Acids Res..

[14] Tang L., Morris J., Wan J., Moore C., Fujita Y., Gillaspie S. (2017). Competition between translation initiation factor eIF5 and its mimic protein 5MP determines non-AUG initiation rate genome-wide. Nucleic Acids Res..

[15] Singh C.R., Watanabe R., Zhou D., Jennings M.D., Fukao A., Lee B. (2011). Mechanisms of translational regulation by a human eIF5-mimic protein. Nucleic Acids Res..

[16] Huang L., Chen S., Fan H., Ai F., Sheng W. (2020). BZW2 promotes the malignant progression of colorectal cancer *via* activating the ERK/MAPK pathway. J. Cell. Physiol..

[17] Ge J., Mu S., Xiao E., Tian G., Tao L., Li D. (2022). Expression, oncological and immunological characterizations of BZW1/2 in pancreatic adenocarcinoma. Front. Genet..

[18] Wang Y.Q., Wu D.H., Wei D., Shen J.Y., Huang Z.W., Liang X.Y. (2023). TEAD4 is a master regulator of high-risk nasopharyngeal carcinoma. Sci. Adv..

[19] Chen L., Jin C., Liu H., Feng R., Li Z., Zhang J. (2021). Analysis of the role of Ly-1 antibody reactive in different cancer types. Bioengineered.

[20] Hu B., Huang M., Tao L., Li Y., Kuang Y., Liu G. (2023). Mesenchymal stem cells-derived exosomal miR-653-5p suppresses laryngeal papilloma progression by inhibiting BZW2. Clinics (Sao Paulo).

[21] Chen H., Liu H., Qing G. (2018). Targeting oncogenic Myc as a strategy for cancer treatment. Signal Transduct. Target. Ther..

[22] Su D., Kosciuk T., Yang M., Price I.R., Lin H. (2021). Binding affinity determines substrate specificity and enables discovery of substrates for N-myristoyltransferases. ACS Catal..

[23] Zhang Y., Su D., Dzikovski B., Majer S.H., Coleman R., Chandrasekaran S. (2021). Dph3 enables aerobic diphthamide biosynthesis by donating one iron atom to transform a [3Fe-4S] to a [4Fe-4S] cluster in dph1-dph2. J. Am. Chem. Soc..

[24] Ong S.E., Blagoev B., Kratchmarova I., Kristensen D.B., Steen H., Pandey A. (2002). Stable isotope labeling by amino acids in cell culture, SILAC, as a simple and accurate approach to expression proteomics. Mol. Cell. Proteomics.

[25] Zhang X., Cao J., Miller S.P., Jing H., Lin H. (2018). Comparative nucleotide-dependent interactome analysis reveals shared and differential properties of KRas4a and KRas4b. ACS Cent. Sci..

[26] Bhawal R., Fu Q., Anderson E.T., Gibson G.E., Zhang S. (2021). Serum metabolomic and lipidomic profiling reveals novel biomarkers of efficacy for benfotiamine in alzheimer’s disease. Int. J. Mol. Sci..

[27] Stauffer W., Sheng H., Lim H.N. (2018). EzColocalization: an ImageJ plugin for visualizing and measuring colocalization in cells and organisms. Sci. Rep..

[28] Mossman K., Mossman (2017).

[29] Szabadkai G., Bianchi K., Várnai P., De Stefani D., Wieckowski M.R., Cavagna D. (2006). Chaperone-mediated coupling of endoplasmic reticulum and mitochondrial Ca2+ channels. J. Cell Biol..

[30] Rampelt H., Zerbes R.M., van der Laan M., Pfanner N. (2017). Role of the mitochondrial contact site and cristae organizing system in membrane architecture and dynamics. Biochim. Biophys. Acta Mol. Cell Res..

[31] Cárdenas C., Miller R.A., Smith I., Bui T., Molgó J., Müller M. (2010). Essential regulation of cell bioenergetics by constitutive InsP3 receptor Ca2+ transfer to mitochondria. Cell.

[32] Pierro C., Cook S.J., Foets T.C.F., Bootman M.D., Roderick H.L. (2014). Oncogenic K-Ras suppresses IP3-dependent Ca2+ release through remodelling of the isoform composition of IP3Rs and ER luminal Ca2+levels in colorectal cancer cell lines. J. Cell Sci,.

[33] Colombini M. (2004). VDAC: the channel at the interface between mitochondria and the cytosol. Mol. Cell. Biochem..

[34] Kornmann B. (2013). The molecular hug between the ER and the mitochondria. Curr. Opin. Cell Biol..

[35] Romero-Garcia S., Prado-Garcia H. (2019). Mitochondrial calcium: transport and modulation of cellular processes in homeostasis and cancer (review). Int. J. Oncol..

[36] Patergnani S., Suski J.M., Agnoletto C., Bononi A., Bonora M., De Marchi E. (2011). Calcium signaling around mitochondria associated membranes (MAMs). Cell Commun. Signal..

[37] Rosencrans W.M., Rajendran M., Bezrukov S.M., Rostovtseva T.K. (2021). VDAC regulation of mitochondrial calcium flux: from channel biophysics to disease. Cell Calcium.

[38] Hajn6czky G., Robb-Gaspers L.D., Seitz M.B., Thomas A.P. (1995). Decoding of C osolic calcium oscillations in the mitochondria. Cell.

[39] Chen T.W., Wardill T.J., Sun Y., Pulver S.R., Renninger S.L., Baohan A. (2013). Ultrasensitive fluorescent proteins for imaging neuronal activity. Nature.

[40] Finkel T., Menazza S., Holmström K.M., Parks R.J., Liu J., Sun J. (2015). The ins and outs of mitochondrial calcium. Circ. Res..

[41] Rossi A., Pizzo P., Filadi R. (2019). Calcium, mitochondria and cell metabolism: a functional triangle in bioenergetics. Biochim. Biophys. Acta Mol. Cell Res..

[42] Gherardi G., Monticelli H., Rizzuto R., Mammucari C. (2020). The mitochondrial Ca2+ uptake and the fine-tuning of aerobic metabolism. Front. Physiol..

[43] Mathupala S.P., Ko Y.H., Pedersen P.L. (2009). Hexokinase-2 bound to mitochondria: cancer’s stygian link to the ‘Warburg Effect’ and a pivotal target for effective therapy. Semin. Cancer Biol..

[44] Dimmer K.S., Rapaport D. (2017). Mitochondrial contact sites as platforms for phospholipid exchange. Biochim. Biophys. Acta Mol. Cell Biol. Lipids.

[45] Coleman R.A., Mashek D.G. (2011). Mammalian triacylglycerol metabolism: synthesis, lipolysis, and signaling. Chem. Rev..

[46] Friedman J.R., Mourier A., Yamada J., Michael McCaffery J., Nunnari J. (2015). MICOS coordinates with respiratory complexes and lipids to establish mitochondrial inner membrane architecture. Elife.

[47] Barneda D., Cosulich S., Stephens L., Hawkins P. (2019). How is the acyl chain composition of phosphoinositides created and does it matter?. Biochem. Soc. Trans..

[48] Li H., Feng Z., He M.-L. (2020). Lipid metabolism alteration contributes to and maintains the properties of cancer stem cells. Theranostics.

[49] Heiden M.G.V., Cantley L.C., Thompson C.B. (2009). Understanding the warburg effect: the metabolic requirements of cell proliferation. Science.

[50] Rodríguez C., Muñoz M., Contreras C., Prieto D. (2021). AMPK, metabolism, and vascular function. FEBS J..

[51] Rodríguez C., Contreras C., Sáenz-Medina J., Muñoz M., Corbacho C., Carballido J. (2020). Activation of the AMP-related kinase (AMPK) induces renal vasodilatation and downregulates Nox-derived reactive oxygen species (ROS) generation. Redox Biol..

[52] Yang Z., Wang L., Yang C., Pu S., Guo Z., Wu Q. (2022). Mitochondrial membrane remodeling. Front. Bioeng. Biotechnol..

[53] de Klerk E., t Hoen P.A.C. (2018). Alternative mRNA transcription, processing, and translation: insights from RNA sequencing. Trends Genet..

[54] Hashem Y., Frank J. (2018). The jigsaw puzzle of mRNA translation initiation in eukaryotes: a decade of structures unraveling the mechanics of the process. Annu. Rev. Biophys..

[55] Sendoel A., Dunn J.G., Rodriguez E.H., Naik S., Gomez N.C., Hurwitz B. (2017). Translation from unconventional 5′ start sites drives tumour initiation. Nature.

[56] Vattem K.M., Wek R.C. (2004). Reinitiation involving upstream ORFs regulates ATF4 mRNA translation in mammalian cells. Proc. Natl. Acad. Sci. U. S. A..

[57] Naghdi S., Hajnóczky G. (2016). VDAC2-specific cellular functions and the underlying structure. Biochim. Biophys. Acta.

[58] Cheng E.H.Y., Sheiko T.V., Fisher J.K., Craigen W.J., Korsmeyer S.J. (2003). VDAC2 inhibits BAK activation and mitochondrial apoptosis. Science.

[59] Yao J., Liu J., Zhao W. (2018). By blocking hexokinase-2 phosphorylation, limonin suppresses tumor glycolysis and induces cell apoptosis in hepatocellular carcinoma. Onco Targets Ther..

[60] Huttlin E.L., Bruckner R.J., Navarrete-Perea J., Cannon J.R., Baltier K., Gebreab F. (2021). Dual proteome-scale networks reveal cell-specific remodeling of the human interactome. Cell.

[61] Taipale M., Tucker G., Peng J., Krykbaeva I., Lin Z.Y., Larsen B. (2014). A quantitative chaperone interaction network reveals the architecture of cellular protein homeostasis pathways. Cell.

[62] Huttlin E.L., Bruckner R.J., Paulo J.A., Cannon J.R., Ting L., Baltier K. (2017). Architecture of the human interactome defines protein communities and disease networks. Nature.

[63] Perez-Riverol Y., Bai J., Bandla C., García-Seisdedos D., Hewapathirana S., Kamatchinathan S. (2022). The PRIDE database resources in 2022: a hub for mass spectrometry-based proteomics evidences. Nucleic Acids Res..

