# Supplementary material for: Interactome Analysis Identifies the Role of BZW2 in Promoting Endoplasmic Reticulum-Mitochondria Contact and Mitochondrial Metabolism
Source: Mol Cell Proteomics. 2023 Dec 26;23(2):100709. doi: 10.1016/j.mcpro.2023.100709 (PMC10835002; doi:10.1016/j.mcpro.2023.100709)
Supplement: Supplemental Figures S1–S7 and Tables S1 and S2 legend [file mmc1.docx]

**Supplementary Figures**

**Figure S1.** (**A**) Western blot images showing the expression level of the no-tagged and Flag-tagged BZW2 and IP efficiency of the FLAG IP. (**B**) Immunoprecipitation of active (Q72L) and inactive (S28N) RalA and RalB shows endogenous BZW2 strongly interacts with the RalA more strongly than RalB in HEK293 cells.

**
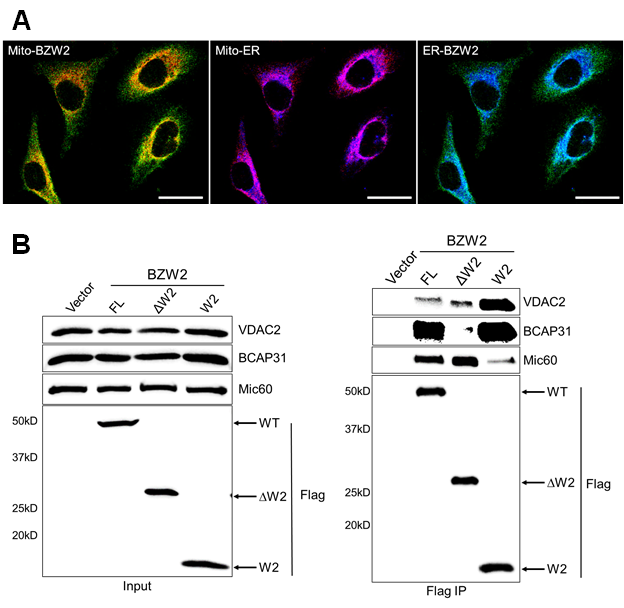
**

**Figure S2.** (**A**) Representative confocal images showing the colocalization of endogenous BZW2, ER (Calnexin) and mitochondria (VDAC) in AsPC-1 cells. Magnification: 63x oil; Scale bar: 22 µm. (**B**) Immunoprecipitation of Flag-tagged full-length BZW2 (FL), BZW2∆W2 (W2 domain deletion), and W2 domain only in HEK293 cells. Deletion of the W2 domain increases BZW2 interaction with the IMM (Mic60) and OMM (VDAC2) while preventing interaction with the ER (BCAP31). The W2 domain of BZW2 alone shows a significant increase in interaction with OMM and ER, yet a significant decrease in IMM interaction.


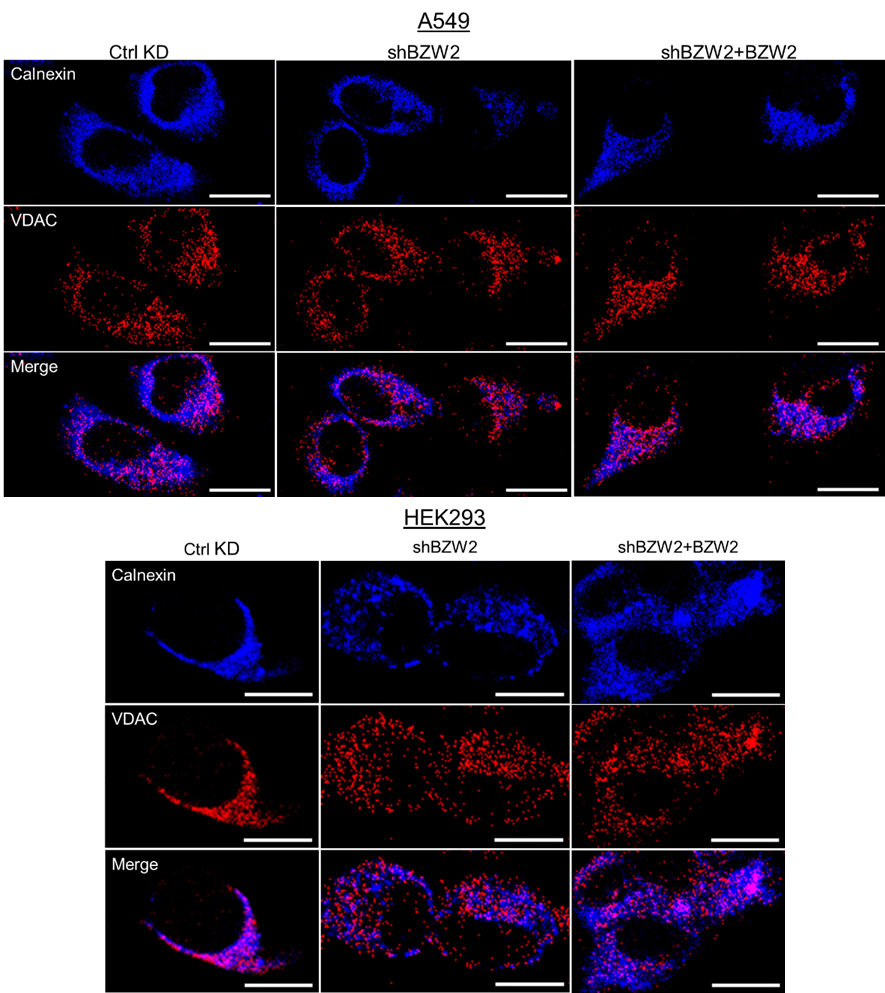


**Figure S3.** Representative confocal images showing the colocalization of ER (Calnexin) and mitochondria (VDAC) in A549 (top panel) and HEK293 (bottom panel) cells with transient BZW2 KD and overexpression. Magnification: 63x oil; Scale bar: 22 µm.

**Figure S4.** (**A**) Visual comparison of mitochondrial staining using Rhod-2AM, reduced Rhod-2AM, Mito4x-GCaMP6f, and Mitotracker. (**B**) (top panel) Western blot showing the KD and re-expression of BZW2 in HEK293 cells. (bottom panel) Representative confocal images of mitochondrial calcium levels using Rhod-2 AM with no treatment (Basal) and after treatment with Ionomycin in live HEK293 cells with transient BZW2 KD and re-expression. Magnification: 40x water; Scale bar: 22 µm. (**C**) (left panel) Representative confocal images of mitochondrial calcium levels using reduced Rhod-2AM (top) and Mito4x-GCaMP6f (bottom panel) with no treatment (Basal) and after treatment with Ionomycin in live HEK293 cells with transient BZW2 KD and re-expression. Magnification: 40x water; Scale bar: 22 µm. (Right panel) Statistical analyses of mitochondrial calcium flux using maximum fluorescence intensity (F_max_)/initial fluorescence intensity (F_0_) show a decrease in mitochondrial calcium flux when BZW2 is knocked down and an increase when BZW2 is re-introduced in live HEK293cells (reduced Rhod-2AM n≥61; Mito4x-GCaMP6f n≥22 ). *p<0.05, **p<0.01, ****p<0.0001.

**Figure S5.** (**A**) Western blot showing stable knockdown of BZW2 in HT-29, SW620 and HEK293 cells. (**B**) Western blot showing the isolated mitochondria and cytosolic fractions of HEK293 cells shows a significant decrease in HKII localization to mitochondria when BZW2 is knocked down. AGK is shown as an additional mitochondrial protein reference (second trial). (**C**) Representative confocal images showing the colocalization of Hexokinase II (HKII) and mitochondria (VDAC) in HEK293 cells with stable BZW2 KD. Magnification: 63x oil; Scale bar: 22 µm.

**Figure S6.** (**A**) Table showing the p-value of the FC in each lipid class when BZW2 is knocked down in HEK293 cells (log2(FC) shown on the right). If sample one is treated as an outlier, the p-value for PE changes from 0.062 to 0.004. (**B**) Heatmap of the log2 of the fold change (FC) in total abundance for each lipid class with a p-value < 0.05 when sample 1 is treated as an outlier in BZW2 KD HEK293 cells. (**C**) Western blot showing an increase in AMPK phosphorylation (T172) when BZW2 is knocked down and a return to normal levels when BZW2 is reintroduced in A549 cells.

**Figure S7.** (**A**) Western blot showing BZW2 KD decreases AKT phosphorylation (T308 and S473) and decreases c-Myc expression in HEK293 cells (two biological replicates). (**B**) Soft agar assay showing BZW2 KD inhibits anchorage independent growth in HCT116 using 5 different BZW2 shRNA’s. The Western blot for BZW2 KD using 5 different shRNA’s in HCT116 cells is shown in top right panel.

**Supplementary Table S1 – SILAC Results**

**Supplementary Table S2 – Lipidomics Results**
